# A spatiotemporal cell atlas of cardiopulmonary progenitor cell allocation during development

**DOI:** 10.1016/j.celrep.2025.115513

**Published:** 2025-04-02

**Authors:** Hongbo Wen, Prashant Chandrasekaran, Annabelle Jin, Josh Pankin, MinQi Lu, Derek C. Liberti, Jarod A. Zepp, Rajan Jain, Edward E. Morrisey, Sylvia N. Michki, David B. Frank

**Affiliations:** 1 Department of Pediatrics, Division of Cardiology, University of Pennsylvania, Children’s Hospital of Philadelphia (CHOP), Penn-CHOP Lung Biology Institute, Penn Cardiovascular Institute, CHOP Cardiovascular Institute, Philadelphia, PA 19104, USA; 2 Department of Pediatrics, Division of Pulmonary and Sleep Medicine, University of Pennsylvania, CHOP, Penn-CHOP Lung Biology Institute, Philadelphia, PA 19104, USA; 3 Department of Medicine, Department of Cell and Developmental Biology, Penn Cardiovascular Institute, Institute for Regenerative Medicine, Perelman School of Medicine at the University of Pennsylvania, Philadelphia, PA 19104, USA; 4 Department of Medicine, Department of Cell and Developmental Biology, Penn-CHOP Lung Biology Institute, Penn Cardiovascular Institute, Institute for Regenerative Medicine, Perelman School of Medicine at the University of Pennsylvania, Philadelphia, PA 19104, USA; 5 These authors contributed equally; 6 Lead contact

## Abstract

The heart and lung co-orchestrate their development during organogenesis. The mesoderm surrounding both the developing heart and anterior foregut endoderm provides instructive cues guiding cardiopulmonary development. Additionally, it serves as a source of cardiopulmonary progenitor cells (CPPs) expressing *Wnt2* that give rise to both cardiac and lung mesodermal cell lineages. Despite the mesoderm’s critical importance to both heart and lung development, mechanisms guiding CPP specification are unclear. To address this, we lineage traced *Wnt2*^+^ CPPs at E8.5 and performed single-cell RNA sequencing on collected progeny across the developmental lifespan. Using computational analyses, we created a CPP-derived cell atlas that revealed a previously underappreciated spectrum of CPP-derived cell lineages, including all lung mesodermal lineages, ventricular cardiomyocytes, and epicardial and pericardial cells. By integrating spatial mapping with computational cell trajectory analysis and transcriptional profiling, we have provided a potential molecular and cellular roadmap for cardiopulmonary development.

## In brief

Using a combination of *in vivo* lineage tracing and single-cell RNA sequencing, Wen et al. have demonstrated an innovative method for studying early cardiopulmonary cell fate determination in addition to providing significant insights into the molecular programs guiding early heart and lung development.

## Graphical abstract

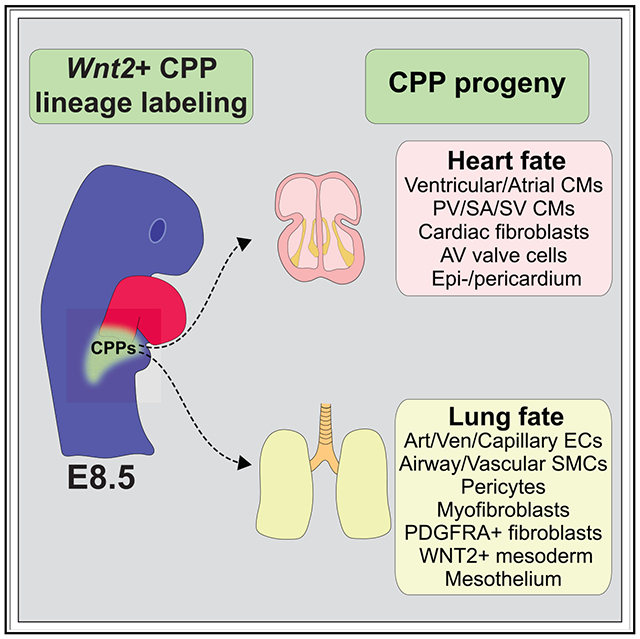

## INTRODUCTION

During organogenesis, co-development of the heart and lung is mediated by a mesodermal cell population that envelopes the area between the heart and lung. This cardiopulmonary mesoderm performs multifunctional roles in cardiopulmonary development. Importantly, it provides the first signaling instructions for lung bud formation and outgrowth.^1–3^ In addition, a subset of this mesoderm comprises the secondary heart field (SHF) that predominantly patterns the right ventricle, the atria, and outflow tract.^4,5^ Similarly, a posterior portion of the SHF containing cardiopulmonary progenitor cells (CPPs) can give rise to cardiomyocytes (CMs) of the cardiac inflow tract, the entire cellular composition of the proximal intrapulmonary pulmonary veins and arteries, and additional mesodermal derivatives of the lung.^3,6,7^

CPPs are marked by *Wnt2*, *Gli1*, or *Isl1* expression and arise around embryonic day (E) 8.5 in a portion of the cardiac mesoderm and splanchnic mesoderm, ultimately giving rise to CMs of the atrium and sinus venosus (SV) as well as all layers of the proximal intrapulmonary arteries and veins, pericytes, and airway smooth muscle (ASM) cells (SMCs).^6,7^ Thus far, molecular mechanisms directing CPP fate are limited and include sonic hedgehog signaling.^6,8^ While many studies have linked signaling mechanisms such as WNT signaling for development of the heart or lung individually, common pathways instructing heart or lung cell fate decisions are less clear.^1,7,9–22^ Furthermore, what guides the spatiotemporal specification for increasingly more diverse cell lineages is unclear.

Using recently developed high-throughput technologies such as single-cell RNA sequencing (scRNA-seq), studies have generated cell atlases of the developing lung and heart. In the heart, transcriptional profiling of single cells has primarily focused on early organogenesis and has uncovered mechanisms of early cell fate specification of cardiac progenitor cells.^23–33^ These include the specification of the second heart field that is contiguous if not overlapping with mesoderm in the area of the developing lung. Similarly, cell atlases of the developing lung have helped identify lung mesodermal cell types and the pathways involved in differentiation and growth.^34–40^ A significant majority of these studies do not ascertain the spatiotemporal cell fate of the earliest stages of lung development. However, a recent study profiled early lateral plate and splanchnic mesoderm surrounding the anterior foregut and identified early specification of foregut mesoderm into its organ-specific constituents.^8^ While these data provided insight into the spatiotemporal mechanisms of cardiopulmonary mesoderm specification, the molecular mechanisms driving specification of cardiac and pulmonary mesodermal lineages remain unclear.

To assess CPP spatiotemporal contribution to heart and lung development, we performed scRNA-seq on *Wnt2*^+^ CPPs progeny labeled at E8.5 over various time points during development. Interestingly, our data indicate a previously underappreciated spectrum of CPP-derived cell lineages, including all mesodermal lineages in the lung, ventricular CMs, pericardial, epicardial, and mesothelial cells. Using computational trajectory analyses and integrated data from multiple datasets, we inferred and characterized the early specification of these lineages from a heterogeneous pool of CPPs. Moreover, transcription factor profiling of individual cell compartments predicted unique and common transcription factors and effectors that may be involved in the specification of these populations. Finally, scRNA-seq uncovered modest vascular hypoplasia and the potential downstream mechanisms associated with loss of WNT2 ligand. In summary, this study provides a novel, comprehensive cell atlas of CPP-derived lineage specification and a framework for assessing molecular mechanisms of spatiotemporal allocation of CPPs.

## RESULTS

### scRNA-seq defines CPP allocation into heart and lung lineages

**Figure 1.**
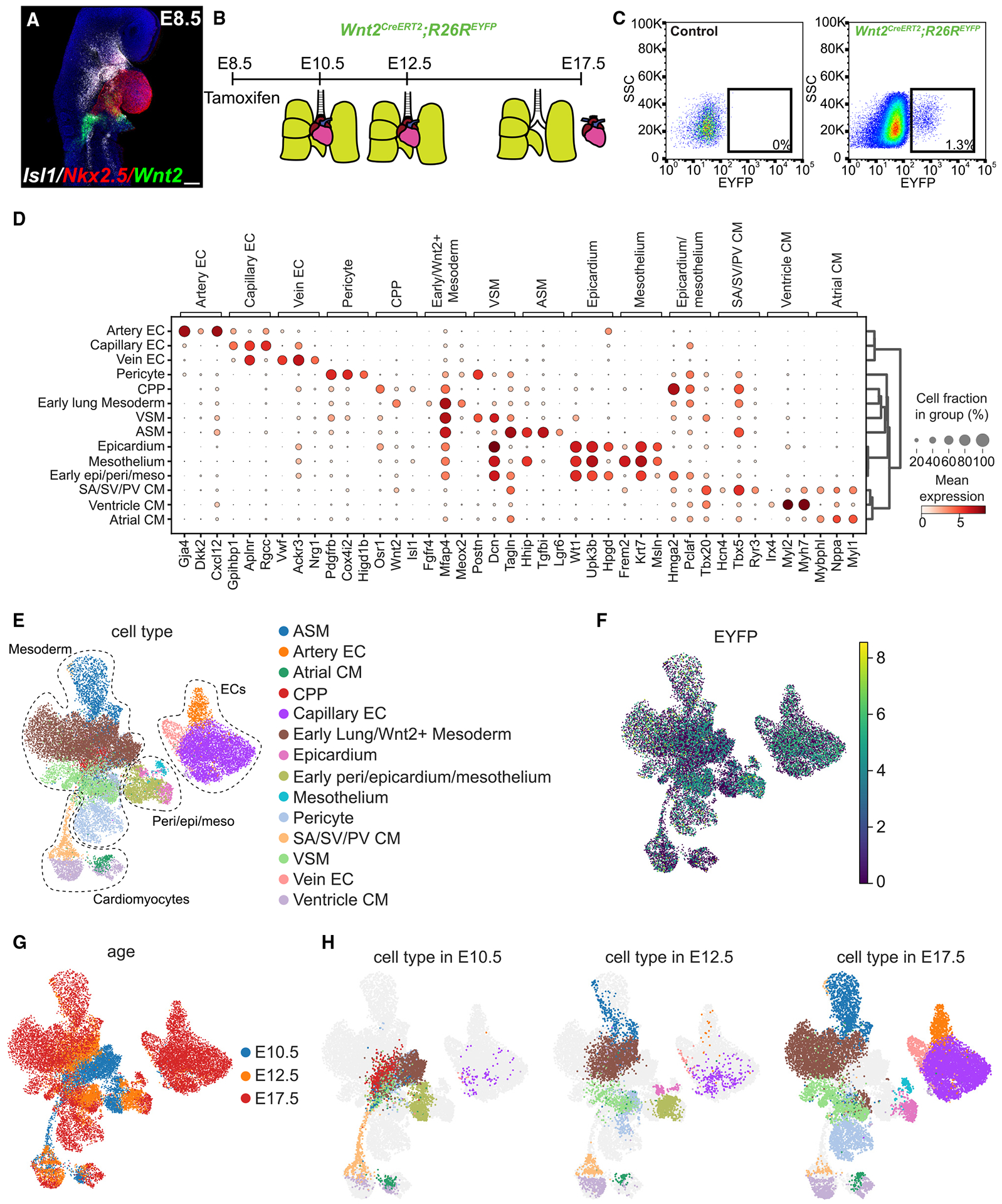
Generation of a spatiotemporal cell atlas of *Wnt2*^+^ CPP progeny across development (A) Whole-mount RNA FISH of E8.5 embryo for *Isl1*/*Nkx2-5*/*Wnt2*. Scale bar, 100 μm. (B) Strategy for tamoxifen administration at E8.5 and tissue collection and cell sorting at E10.*5*, E12.5, and E17.5. (C) Fluorescence-activated cell sorting (FACS) strategy for isolation of EYFP^+^ cells in control (left) and *Wnt2*^*CreERT2*^*;R26R*^*EYFP*^ (right) embryos. (D) Dot plot of canonical marker genes for each cell type, where dot size and color indicate the cell proportion and expression level, respectively, within the cluster. (E) UMAP embedding colored by cell types. (F) UMAP embedding colored by EYFP expression. (G) UMAP embedding colored by age. (H) UMAP embedding colored by cell types represented at E10.5 (left), E12.5 (center), and E17.5 (right).

To determine the contribution of CPPs in heart and lung development, we performed RNA fluorescent *in situ* hybridization (FISH) to determine Wnt2 expression domains at E8.5. Using a combination of probes, including *Wnt2*, *Isl1*, and *Nkx2-5*, we confirmed robust *Wnt2* expression in the area of the posterior portion or venous pole of the cardiac mesoderm into the foregut splanchnic mesoderm (Figure 1A). In addition, we stained for *Wnt2*, *Nkx2-5*, and *Hoxb1* and confirmed co-expression of *Wnt2* and *Hoxb1* in the posterior portion of the SHF as previously described, but *Hoxb1* expression is broader extending toward the anterior portion of the SHF and extending inferior of the *Wnt2* expression domain (Figure S1A).^41^

We induced recombination at E8.5 in pregnant *Wnt2*^+*/CreERT*2^*; R26R*^*EYFP*^ females with tamoxifen intraperitoneal injections. We prepared single-cell suspensions of combined heart and lung enhanced yellow fluorescent protein-positive (EYFP^+^) cells at E10.5 and E12.5 and separated heart and lung EYFP^+^ cells at E17.5 using fluorescence-activated cell sorting (FACS) for live cells expressing EYFP (Figures 1B and 1C). From three time points, we profiled 24,526 cells after filtering out dying and low-quality cells based on quality assessment metrics. Using unbiased and canonical marker gene approaches, we performed dimensionality reduction, batch correction, and Leiden clustering on the integrated datasets. We identified 20 distinct cell clusters and 14 annotated cell types (Figures 1D, 1E, S1B, and S1C). From these analyses, we identified the presence of not only previously noted CPP-derived populations but also multiple underappreciated cell populations, including lung capillary endothelial cells (ECs) and mesothelial cells, cardiac epicardial and pericardial cells, and ventricular CMs (Figure 1E). Previous studies have indicated that cells of the proepicardial organ give rise to cardiac epicardial and pericardial progeny and are derived from lateral plate mesoderm, which also contributes to the mesodermal heart fields.^42,43^ To determine whether *Wnt2* is expressed in the region of the epicardium and proepicardial organ, we performed RNA FISH for *Wnt2* and *Wt1*, revealing colocalization of cells in the area of posterior region of the SHF and SV (Figures S1D and S1E). All subpopulations expressed *Eyfp* transcripts, confirming that previously identified and new cell populations are derived from *Wnt2*^+^ CPPs (Figure 1F). In addition, major compartments including CMs, endothelium, lung mesoderm, and epicardium/mesothelium were represented at all time points (Figure 1G). Examination of the temporal specification of CPPs demonstrated early specification into heart and lung lineages with increasingly more cell lineages as development progressed (Figure 1H).

### Wnt2^+^ CPPs differentiate into the diverse lineages of the heart

**Figure 2.**
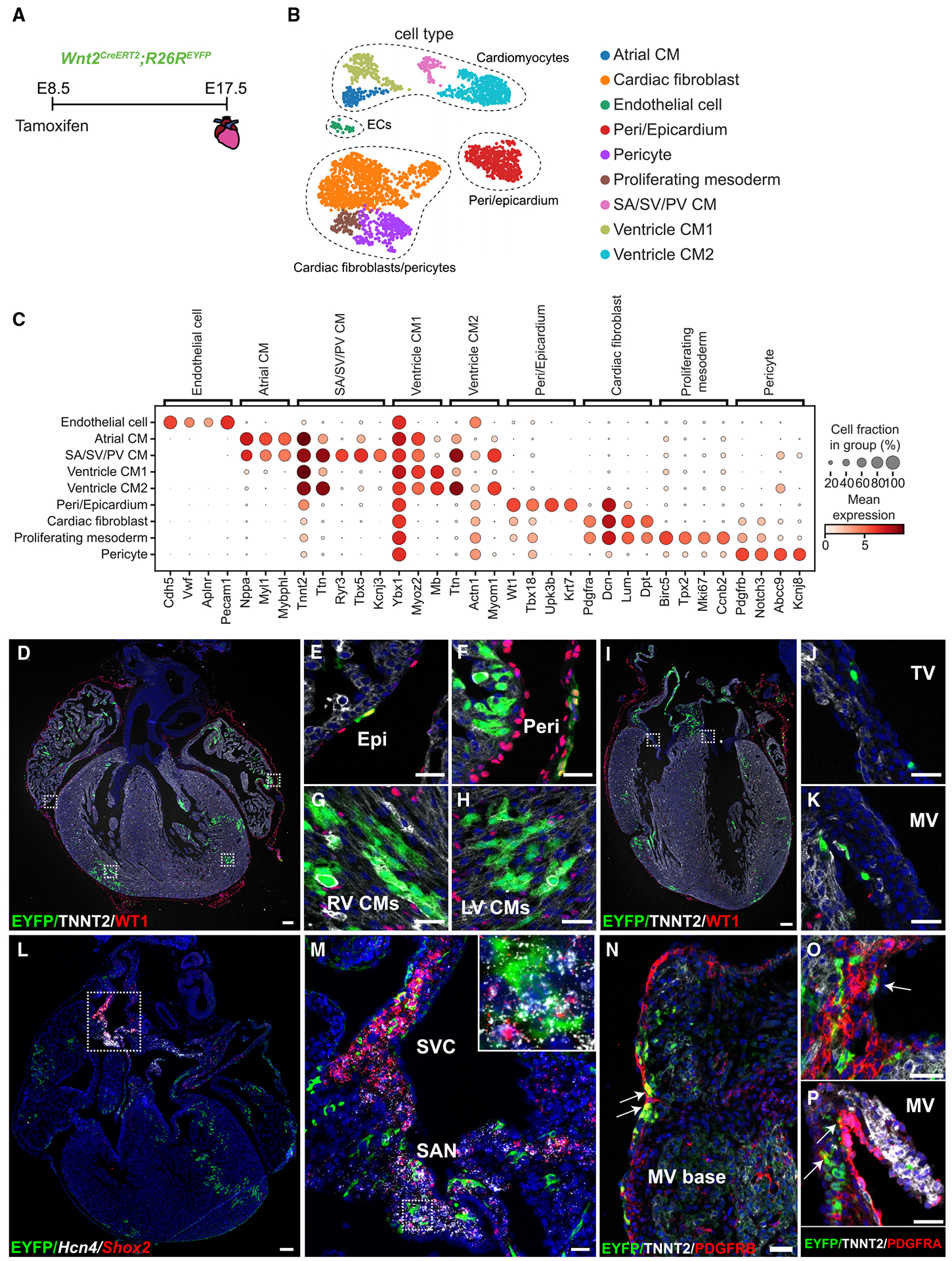
*Wnt2*^+^ CPPs give rise to diverse cell populations in the heart (A) Recombination induction and harvest of hearts at E17.5. (B) UMAPs colored by cell type in the heart. (C) Dot plot of canonical marker genes for cell types, where dot size indicates the cell proportion and dot color represents the expression level, respectively, within the cluster. (D) IHC for TNNT2, WT1, and EYFP at E17.5. (E–H) Higher-magnification IHC from boxed areas in (D) representing (E) epicardium (Epi), (F) pericardium (Peri), (G) right ventricle (RV) cardiomyocytes (CMs), and (H) left ventricle (LV) CMs. (I) IHC for TNNT2, WT1, and EYFP at E17.5. (J and K) Higher-magnification IHC represents boxed areas of (J) tricuspid valve (TV) and (K) mitral valve (MV). (L and M) (L) RNA FISH for *Hcn4* and *Shox2*, with IHC for EYFP. (M) Higher magnification of boxed areas in (L) showing RNA FISH for *Hcn4* and *Shox2*, with IHC for EYFP in the superior vena cava (SVC) and sinoatrial node (SAN). (N) IHC for TNNT2, PDGFRB, and EYFP at E17.5 at the mitral valve (MV) base. (O and P) IHC for TNNT2, PDGFRA, and EYFP at E17.5 at junction of atrium and ventricle (O) and the mitral valve (MV) (P). Scale bars, 100 μm for (D), (I), and (L), and 25 μm for (E)–(H), (J), (K), and (M)–(P).

We extracted data from the E17.5 heart sample to examine more terminally differentiated CPP-derived cells in the heart (Figure 2A). Heart samples consisted of the heart proper and both the pulmonary artery (PA) and the pulmonary vein (PV) up to the hilum of the lung. After cell-type annotation using both unbiased differential gene expression analysis and canonical cell markers, we identified nine cell populations, including ECs, expressing *Cdh5* and *Pecam1*, pericytes expressing *Pdgfrb* and *Abcc9*, fibroblasts expressing *Pdgfra* and *Dpt*, proliferating mesoderm expressing *Mki67* and *Ccnb2*, pericardium/epicardium expressing *Wt1* and *Upk3b*, sinoatrial artery (SA)/SV/PV CMs expressing *Ryr3* and *Kcnj3*, and atrial CMs expressing *Nppa* and *Mybphl* (Figures 2B and 2C). Interestingly, we noted two different ventricular CM populations, labeled ventricular CM1 and CM2. Both populations expressed markers for ventricular CMs such as *Irx4*, *Myl2*, and *Myh7* (Figure S2A). However, compared to CM2, cells in ventricular CM1 exhibited lower expression of genes involved in the cardiac sarcomere and other structural proteins, including *Ttn*, *Actn1*, *Myom1*, *Trdn*, and *Dmd* (Figures 2C, S2A, and S2B), indicating an immature CM subpopulation. This was also supported by a significantly larger proportion of CM1 cells in G2/M and S phase, indicative of a proliferating, immature cell state (Figure S2C). In addition, cells of CM1 had lower expression of markers of cardiac maturation including estrogen receptor gamma (*Esrrg*), which is found almost exclusively in the cells of CM2.^44^ Using an antibody for ESRRG, we observed expression in both right and left ventricular CMs (Figure S2D).^45,46^ Additionally, CM1 expressed lower levels of mature CM markers of compact myocardium, *Hey2*, *Tbx20*, and *Erbb4*, and conduction and calcium handling, including *Scn5a*, *Kcnj2*, *Cacna1c*, *Ryr2*, *Slc8a1*, and *Atp2a2* (Figure S2B).^47,48^ While cells of CM1 did not express a specific marker to localize cells of this cluster, they did express slightly higher levels, *Ybx1*, *Myoz2*, and *Eef1a1*, genes recently identified in a human fetal CM subtype with no specific spatial localization and found throughout the ventricles (Figures 2C and S2B).^49^

To corroborate and spatially define *Wnt2*^+^ CPP-derived cardiac populations, we performed RNA FISH and/or immunohistochemistry (IHC) on E17.5 embryos lineage traced at E8.5. We first performed IHC using the general CM marker TNNT2 and the epicardial marker WT1, combined with GFP antibody staining to identify EYFP^+^ CPP-derived cells. We identified EYFP^+^ cells in the epicardium pericardium, atrium, left ventricle (LV), and right ventricle (RV) regions (Figures 2D–2H). In addition to these regions, we confirmed the presence of *Wnt2*^+^ CPP-derived cells in the tricuspid valve (TV), mitral valve (MV), and vena cava (Figures 2I–2K, S2E, and S2F). We next used RNA FISH combined with IHC to spatially localize other CPP-derived subpopulations. Using a GFP antibody to detect EYFP and RNAscope probes for *Hcn4* and *Shox2*, we identified *Wnt2*^+^ CPP-derived CMs of the SA node in addition to the vena cava and PV, respectively (Figures 2L and 2M). Additionally, we confirmed the presence of *Wnt2*^+^ CPP progeny in both left and right superior vena cava (SVC), inferior vena cava, and coronary sinus (Figures S2G–S2L). Furthermore, there were *Wnt2*^+^ CPP-derived platelet-derived growth factor receptor alpha-positive, PDGFRA^+^ and PDGFR beta-positive (PDGFRB^+^) valve cells and PDGFRA^+^ subepicardial fibroblast-like cells commonly found in the area of the atrial-ventricular junction (Figures 2N–2P and S2M). Of note, EYFP^+^ cells were absent in the endocardium, coronary vasculature, semilunar valves, RV outflow tract, aorta, and the main PA. These findings uncover new CPP-derived populations and provide additional spatial data on CM subtypes.

### *Wnt2*^+^ CPPs give rise to most mesodermal lineages in the lung

**Figure 3.**
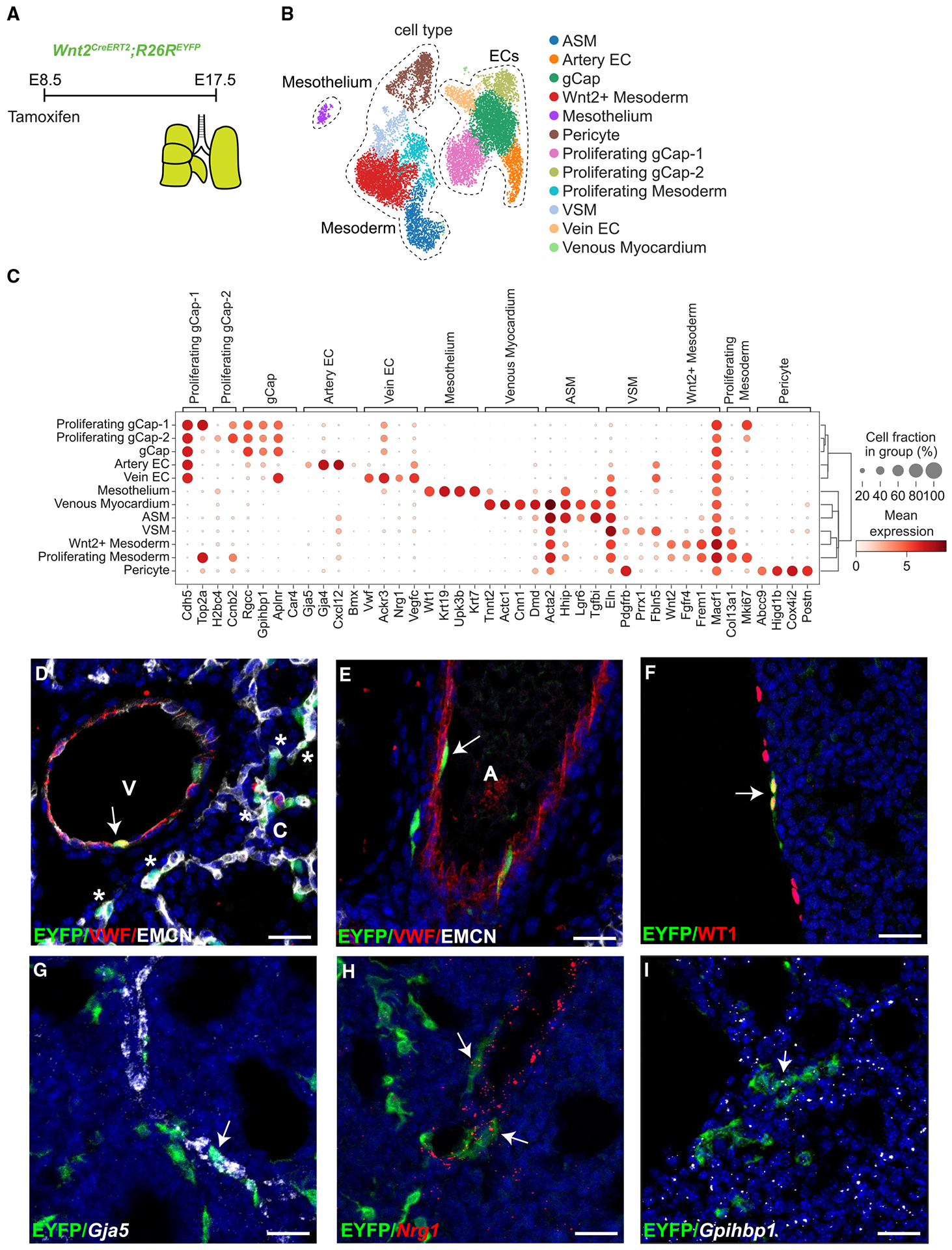
*Wnt2*^+^ CPPs label mesodermal lineages in lung (A) Tamoxifen administration and harvest time point at E17.5 in the lung.. (B) UMAP embedding colored by cell type.. (C) Dot plot consisting of canonical marker genes for cell types, where dot size and color intensity indicate the proportion of cell and gene expression, respectively, within the cluster.. (D and E) IHC staining for VWF, EMCN, and EYFP (A, artery; C, capillaries; V, vein).. (F) IHC for WT1 and EYFP. (G–I) RNA FISH and IHC for (G) *Gja5* and EYFP, (H) *Nrg1* and EYFP, and (I) *Gpihbp1* and EYFP. Scale bars, 25 μm.

We performed similar analyses on the transcriptomic data extracted from the E17.5 lung dataset (Figure 3A). After performing marker gene analysis, we identified 11 cell populations (Figures 3B and 3C). Importantly, we confirmed the presence of distal capillary ECs not observed in previous data on CPPs.^6^ Moreover, capillary ECs were delineated into three separate clusters, primarily related to their phase of the cell cycle (Figure S3A). While the presence of general capillary ECs (gCaps) expressing *Gpihbp1* and *Aplnr* was noted, there were two clusters of proliferating gCaps called gCap-1 and gCap-2 in G2/mitotic phase and/or S phase (Figures 3B and S3A). Alveolar capillary ECs (aCaps) were not positively identified, consistent with their differentiation slightly later in development.^34,36,37,40^ Among other EC subtypes, we confirmed the presence of arterial ECs, marked by the expression of *Gja5* and *Vwf*, and venous ECs expressing *Vwf* and *Nrg1* (Figures 3B and 3C). An additional previously underappreciated mesodermal lung cell type derived from *Wnt2*^+^ CPPs included the mesothelium, marked by the expression of *Wt1* and *Upk3b* (Figures 3B and 3C). Similar to the cardiac subtypes, we confirmed the presence of previously identified cells, including venous myocardium, ASM, vascular smooth muscle (VSM), Wnt2^+^ mesenchyme, pericytes, and proliferating mesoderm (Figures 3B and 3C).

Using IHC or a combination of IHC and RNA FISH, we validated the presence of distal capillary ECs that were positive for the venous EC and capillary EC marker, endomucin (EMCN), and negative for the macrovessel marker, von Willebrand factor (VWF) (Figure 3D). In addition, both venous and arterial ECs were EYFP^+^ (Figures 3D and 3E). Similarly, we were able to confirm the presence of EYFP^+^ and WT1^+^ mesothelium, adding an additional previously unrecognized CPP-derived cell population (Figure 3F). We also performed RNA FISH using probes for markers of specific EC subtypes, including *Gja5* (artery), *Nrg1* (vein), and *Gpihbp1* (capillaries), and were able to corroborate all specific EC subtypes (Figures 3G–3I). Additionally, we confirmed the spatial localization of lung mesenchymal cell lineages such as ASM (ACTA2, *Lgr6*), VSM (VWF, ACTA2, and *Pdgfrb*), venous myocardium (TNNT2), pericytes (*Abcc9* or *Pdgfrb*), and *Wnt2*^+^ mesoderm (*Fgfr4* or *Wnt2*) (Figures S3B–S3G).

While we were able to identify several mesodermal-specific populations, including airway and vascular SMCs and pericytes at E17.5, the remainder of the lung mesodermal populations consisted of a single Wnt2^+^ mesoderm that expressed markers of fibroblast populations found in the lung.^35–38,50,51^ In particular, *Wnt2*, *Pdgfra*, *Plin2*, and *Pdgfrb*/*Acta2*, representing fibroblast progenitors, alveolar fibroblasts, lipofibroblasts, and myofibroblasts, respectively, were also expressed throughout the *Wnt2*^+^ mesodermal population (Figure S3H). Prior studies indicate that *Wnt2*^+^ mesodermal progenitors can give rise to the PDGFRA^+^ alveolar fibroblasts later in development.^38^ To investigate whether we could specifically label more mature and specific fibroblast cell types, we performed lineage tracing of *Wnt2*^+^ CPPs at E8.5 and followed them into adulthood. Using IHC for PDGFRA and PDGFRB, we noted numerous EYFP^+^ cells located in the alveolar interstitium expressing PDGFRB suggestive of pericytes or myofibroblasts and cells expressing PDGFRA indicative of alveolar fibroblasts (Figures S3I and S3J).

### Differential recombination efficiencies in *Rosa26* reporters with *Wnt2*^*CreERT2*^

We have identified previously unrecognized cell types derived from *Wnt2*^+^ CPPs. The previous study performing cell fate mapping at E8.5 used a Rosa26-mTmG (*R26R*^*mTmG*^) reporter allele.^6,52^ In the present study, we have employed the Rosa26-EYFP (*R26R*^*EYFP*^) reporter allele that, anecdotally, has proven a robust reporter system to isolate cells in our prior studies.^51,53–55^ As such, we asked whether reporter allele recombination efficiency played a role in the identification of additional cell types derived from CPPs. To test this, we generated a *Wnt2*^*CreERT2*^ double reporter line (*Wnt2*^*CreERT2*^*; R26R*^*mTmG/EYFP*^) consisting of one allele of mTmG (*R26R*^*mTmG*^) and one allele of EYFP (*R26R*^*EYFP*^) (Figure S4A). After induction of recombination at E8.5, we performed FACS with specific filters for GFP and YFP to determine the number of monomeric (m)GFP^+^ and EYFP^+^ cells in the lungs from a single embryo at E17.5 (Figures S4B–S4G). Quantification of mGFP^+^ and EYFP^+^ cells in each lung revealed a very low efficiency in recombination of the *R26R*^*mTmG*^ allele when compared to the *R26R*^*EYFP*^ allele (4- to-150-fold difference in 4 separate litters) (Figure S4H). In addition, a similar difference existed qualitatively in the hearts of each embryo (Figure S4I).

**Figure 4.**
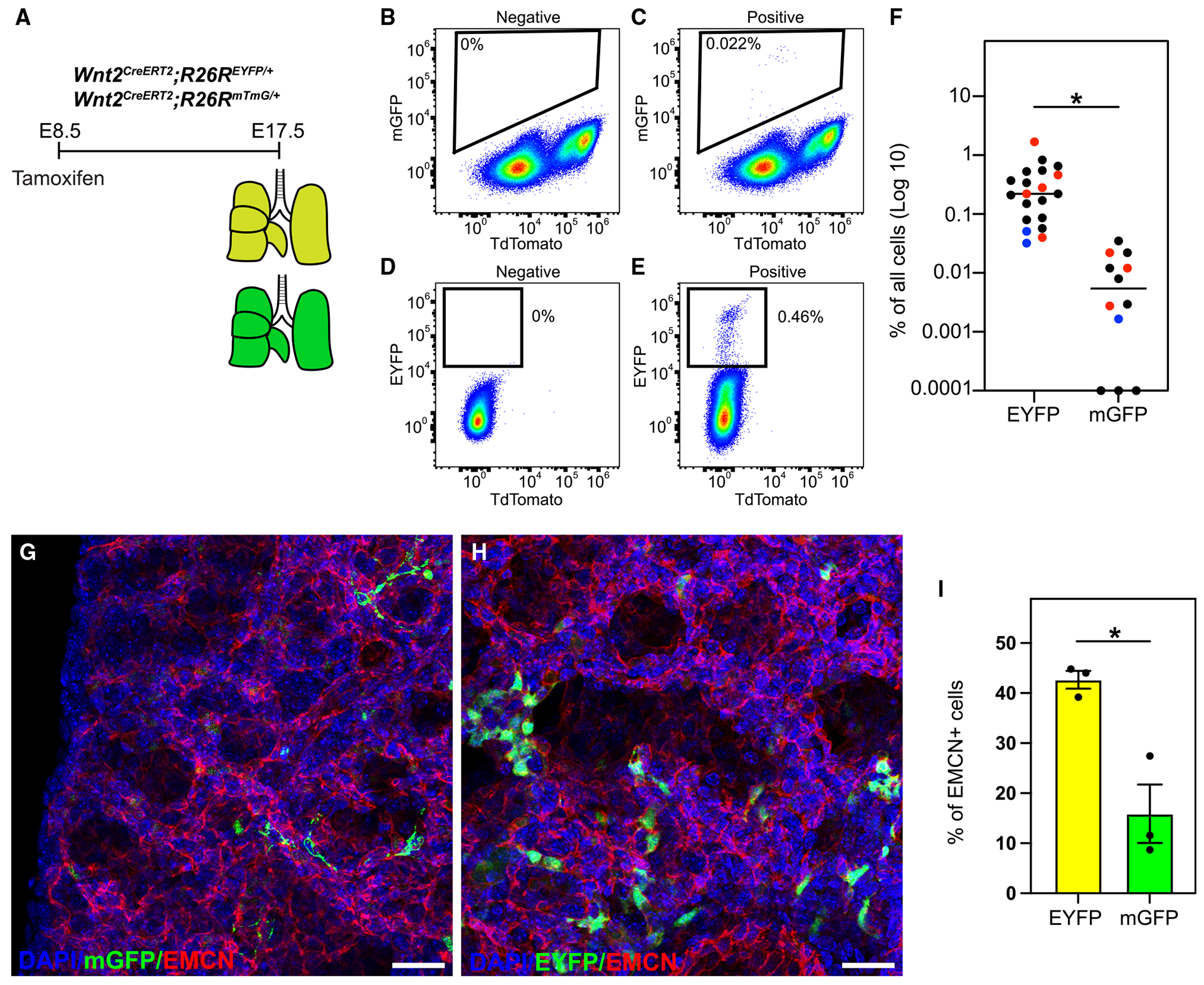
Differential recombination efficiencies of EYFP and mTmG with *Wnt2*^*CreERT2*^ (A) Tamoxifen administration and harvest time point at E17.5 in the lung. (B–E) FACS gating strategy for collection of (B) mGFP^−^ cells, (C) mGFP^+^ cells, (D) EYFP^−^ cells, and (E) EYFP^+^ cells (bottom) in the lung. (F) Percentage of recombination efficiency of EYFP^+^ and mGFP^+^, *n* = 4 litters. Red and blue dots represent two examples of littermates. *p < 0.05; Student’s t test. (G–I) Quantification of distal (capillary) endothelium marked by mGFP or EYFP. (G and H) IHC staining for (G) mGFP and EMCN, and (H) EYFP and EMCN. (I) Percentage of EMCN^+^, EYFP^+^/EYFP^+^ and EMCN^+^, mGFP^+^/mGFP^+^ cells, *n* = 3 embryos. Data are represented as mean ± SEM. *p < 0.05; Student’s t test. Scale bars, 50 μm (G and H).

We saw very little recombination for the *R26R*^*mTmG*^ allele in combination with the *R26R*^*EYFP*^ allele in the same embryo. To rule out the possibility of a bias in recombination for the *R26R*^*EYFP*^ allele in the same embryo, we performed lineage tracing in embryos of the same litter that would contain one copy of either the *R26R*^*mTmG*^ allele or the *R26R*^*EYFP*^ allele (Figure 4A). Flow cytometry analysis was used to distinguish the mGFP^+^ from EYFP^+^ cells (Figures 4B–4E). Consistent with our previous study, we determined, on average for each litter (7 total litters), a 14- to-43-fold increase in cells with the *R26R*^*EYFP*^ allele (Figure 4F). To determine whether this increased recombination also led to an increase in the number of identified distal lung ECs, we quantified the number of EMCN^+^ cells in clusters of mGFP^+^ or EYFP^+^ cells (Figures 4G and 4H). Surprisingly, we did identify mGFP^+^ distal ECs, but there was a nearly 3-fold increase in the number of distal ECs with the *R26R*^*EYFP*^ allele (Figure 4I). Together, these results confirm an increased recombination efficiency using the *R26R*^*EYFP*^ allele.

### *Wnt2*^+^ CPPs initiate cell fate specification early in cardiopulmonary development

**Figure 5.**
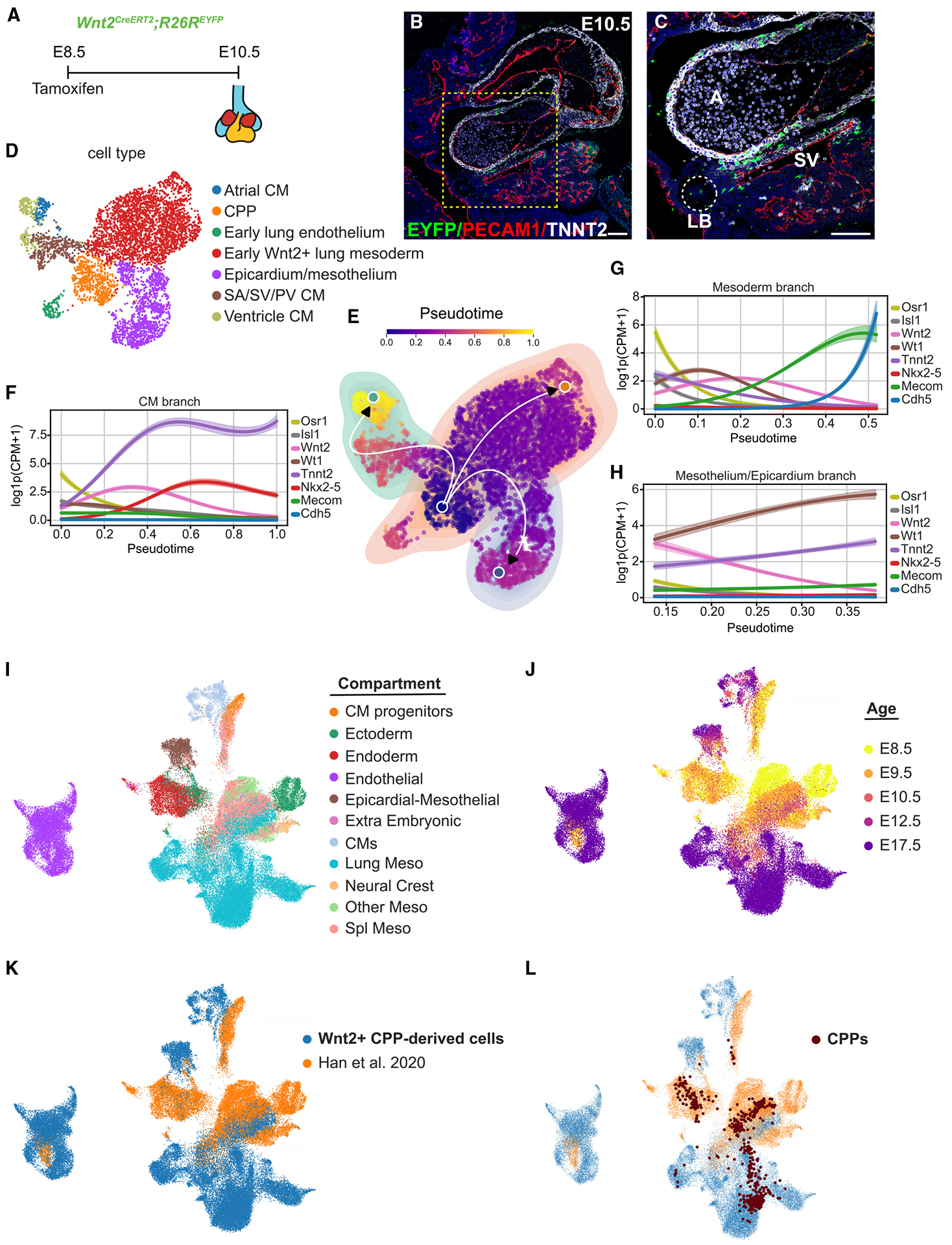
*Wnt2*^+^ CPPs specify cell fate early in cardiopulmonary development (A) Tamoxifen administration and harvest time point of heart and lung at E10.5. (B and C) IHC for (B) PECAM1, TNNT2, and EYFP on E10.5 heart tissue with (C) magnification of boxed area in (B) (A, atrium; LB, lung bud; SV, sinus venosus). Scale bars, 100 μm. (D) UMAP embedded with cells colored by cell type at E10.5. (E–H) (E) Pseudotime trajectory inference applied to cells from E10.5 heart/lung; cells (dots) colored by position along pseudotime; underlay colors indicate inferred branch assignment. (F–H) Gene expression trends for genes known to be involved in lineage specification, fitted across cells belonging to the inferred (F) CM branch, (G) mesoderm branch, and (H) mesothelium/epicardium branch. (I–L) UMAPs generated from integrated data from present study and Han et al.^8^ UMAP embedded with cells colored by (I) cell compartments, (J) time points, (K) datasets used, and (L) datasets with embedded CPPs.

*Wnt2*^+^ CPPs give rise to a diversity of cells that compose the heart and lung. Early studies using clonal lineage tracing revealed that some multipotent CPPs were capable of giving rise to both cardiac and lung lineages.^6^ More recently, a study using scRNA-seq on isolated foregut mesoderm in early organogenesis indicated that splanchnic mesoderm contained separate pools of cardiac and pulmonary progenitors as development progressed.^8^ To determine the temporal allocation of CPP-derived progeny, we performed lineage tracing demonstrating *Wnt2*^+^ CPP cell specification into both cardiac and lung lineages by E10.5 (Figures 5A–5C and S5A). Subsequently, we extracted the lineage-traced E8.5–E10.5 scRNA-seq data and reper-formed single-cell analysis. Uniform manifold approximation and projections (UMAPs) colored by cell types and dot plots were generated using reference gene markers demonstrating specification into seven different cardiac and lung cell populations (Figures 5D and S5B). From these data, we identified early endothelium (*Cdh5*, *Plvap*, *Gimap6*, *Kdr*, *Calcrl*), atrial CMs (*Myl1*, *Nppa*, *Mybphl*, *Tnni2*), ventricle CMs (*Myh7*, *Pln*, *Irx4*, *Hspb7*), SA/SV/PV CMs (*Myom1*, *Myocd*, *Ldb3*, *Fbxo32*, *Ryr3*), epicardium/mesothelium (*Krt7*, *Prkcb*, *Dcn*, *Upk3b*, *Upk1b*), CPPs (*Osr1*, *Foxf1*, *Adgrb3*, *Csmd1)*, and early lung/Wnt2^+^ mesoderm (*Lix1*, *Alx3*, *Gsc*, *Ebf3)* (Figure S5B).

The temporal nature of CPPs enabled us to focus on an early period of development at E10.5 to characterize cell fate decisions. To corroborate early specification, we used pseudotime analysis to infer lineage specification trajectories of *Wnt2*^+^ CPPs at E10.5, identifying three distinct cell fate branches arising from CPPs (Figures 5E–5H). Concomitant with decreasing cell numbers as demonstrated by the reduction in *Osr1* expression, *Wnt2*^+^ CPPs were predicted to be specified along a CM branch indicated by the increased expression of *Tnnt2* and *Nkx2-5* (Figures 5E and 5F), a mesoderm branch identified by the elevated expression of *Mecom* and *Cdh5* (Figures 5E and 5G), and a mesothelium/epicardium branch, marked by increased *Wt1* expression (Figures 5E and 5H).

### CPPs represent a heterogeneous progenitor pool for early development

Previous clonal analysis of *Wnt2*^+^ CPPs indicated that some progenitors gave rise to clones containing both cardiac and lung mesodermal derivatives.^6^ Whether all CPPs exist as a homogeneous population of progenitors is unknown. Recent single-cell transcriptomic evidence suggests that mesoderm surrounding the foregut endoderm is heterogeneous and can be separated into a diverse group of populations, including separate cardiac and lung mesodermal populations by E9.5.^8^ Our single-cell data reveal *Wnt2*^+^ CPPs as transcriptomically similar and segregated as a single population of cells at E10.5 (Figure 5D). However, early transcription programs for specification may be downregulated at this stage of development. To determine whether we could segregate CPPs into these early mesodermal populations, we integrated our scRNA-seq data with E8.5 and E9.5 scRNA-seq data from Han et al.^8^ Using cell annotation methods, we identified 11 major cell populations (Figure 5I). We highlighted the datasets by generating UMAPs colored by time point and by each separate dataset (Figures 5J and 5K). Interestingly, the two experiments had some cells with transcriptomic similarity, but they predominantly separated into transcriptionally distinct cells. However, when we used marker genes to identify CPPs, they were found in multiple distinct clusters of progenitors, including general mesoderm, lung mesoderm, mesothelial/epicardial, and cardiac progenitors, suggesting some heterogeneity in this progenitor population (Figure 5L).

### Differential gene expression defines CPP-derived populations

Our analyses have uncovered several previously underappreciated cell populations and corroborated CPP cell fate. To define transcription factor programs guiding CPP cell fate, we performed cell compartmental differential gene expression analysis on scRNA-seq data from E10.5, E12.5, and E17.5 lineage-traced CPP progeny (Figures S6A–S6C). During early development at E10.5, transcription factor enrichment was apparent in all of the early progenitor populations (Figure S6A). *Wnt2*^+^ CPPs expressed high levels of *Osr1*, *Foxf1*, *Gata5*, and *Tshz1* transcription factors compared to their progeny at E10.5. However, by E12.5, CPPs were absent, and each cell compartment maintained a unique repertoire of transcription factors throughout development (Figures S6B and S6C). Important transcription factors and effectors expressed during development included but were not limited to *Sox17* and *Sox18* for endothelium, *Ppargc1* and *Smyd1* for myocardium, *Tbx4* for mesoderm, and *Wt1* and *Myrf1* for epicardium/mesothelium.

**Figure 6.**
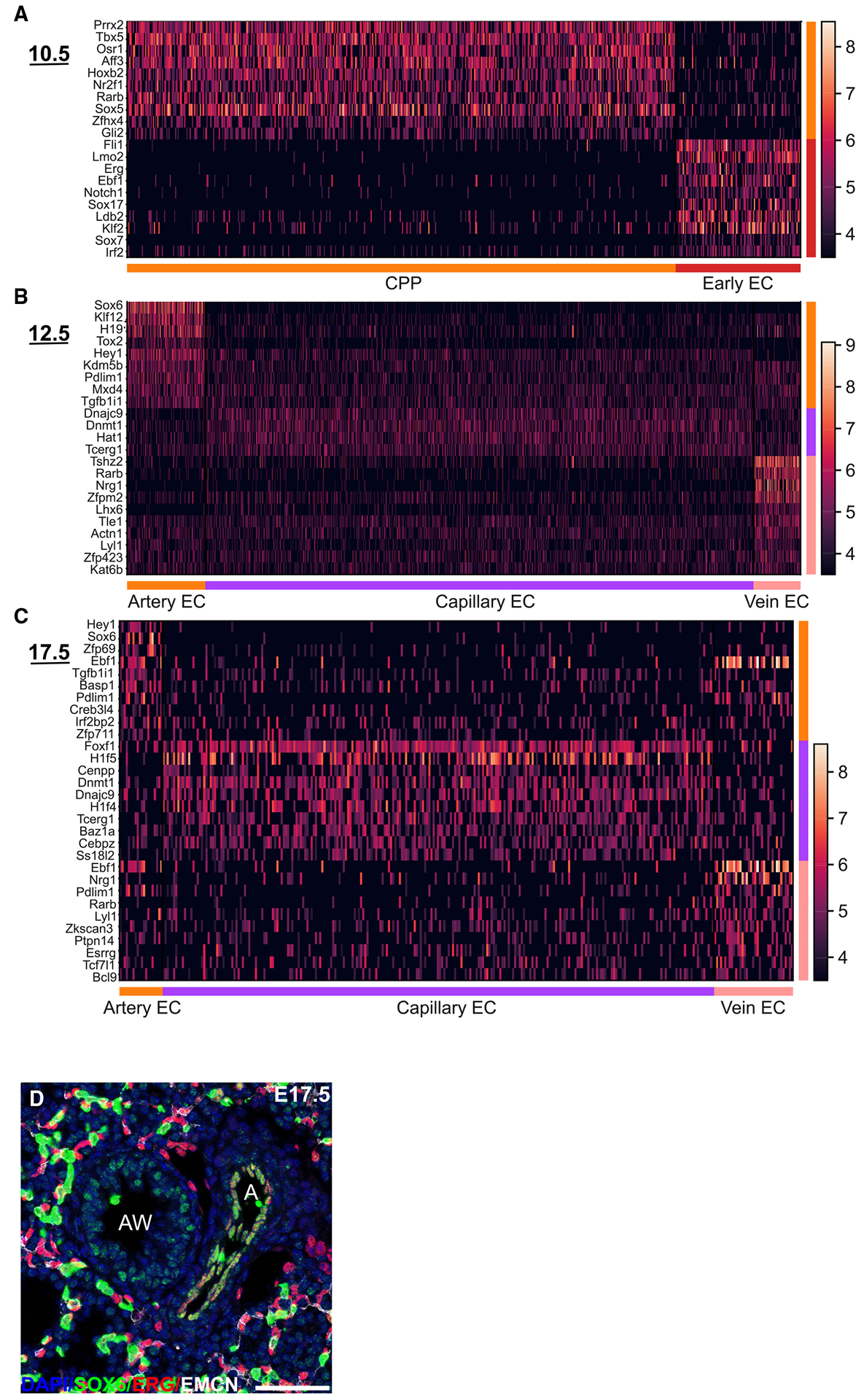
Differential gene expression defining CPP-derived populations (A–C) Heatmaps generated by differential gene expression for (A) CPPs and early ECs at E10.5. (B and C) Arterial, capillary, and venous EC subtypes at (B) E12.5 and (C) E17.5. (D) IHC for SOX6, ERG, and EMCN at E17.5. A, artery; AW, airway. Scale bar, 50 μm.

We further characterized the data by analyzing potential transcription factors associated with specification into arterial, capillary, or venous EC lineages. Heatmaps generated by differential gene expression identified unique transcriptional profiles for each EC subtype (Figures 6A–6C). While there was temporal expression for most transcription factors, EC subpopulations also retained specific transcription factor or DNA-binding protein expression throughout development and included *Sox6* and *Hey1* for arterial ECs, *Lyl1* and *Rarb* for venous ECs, and *Dnajc9* and *Dnmt1* for capillary ECs. To confirm arterial identity associated with specific transcription factors, we performed IHC for SOX6 on E17.5 embryonic lung tissue sections and noted arterial EC-specific SOX6 expression (Figure 6D).

### Ventricular hypoplasia and saccular/capillary simplification in *Wnt2* loss-of-function mutants

**Figure 7.**
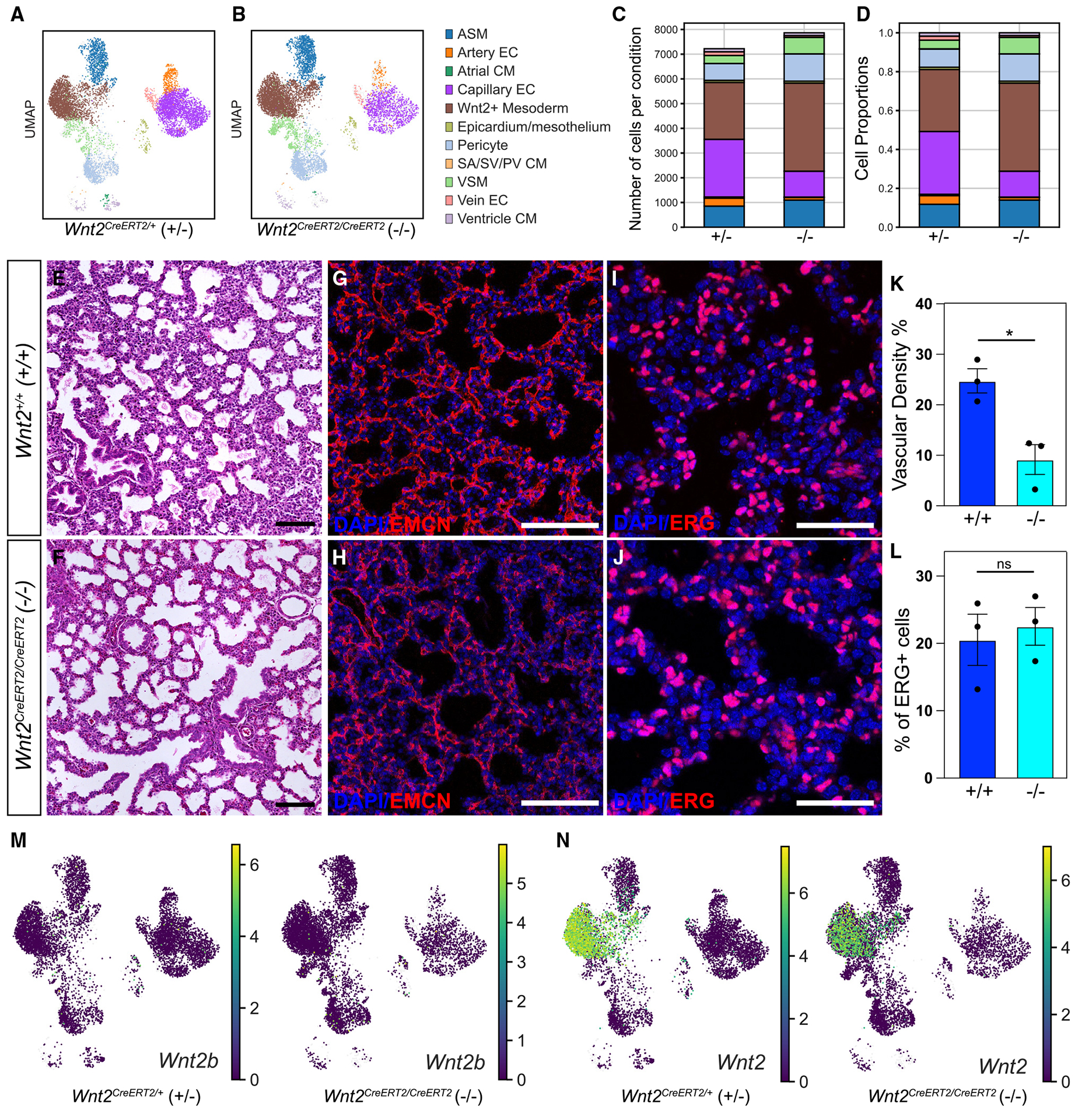
scRNA-seq identifies vascular hypoplasia in *Wnt2*-deficient mice (A and B) UMAP embedding of E17.5 heart and lung EYFP^+^ cells colored by cell type in (A) heterozygous control (*Wnt2*^*CreERT2/+*^) and (B) homozygous mutant (*Wnt2*^*CreERT2/CreERT2*^) mice. (C) Number of EYFP^+^ cells in each identified cell type between *Wnt2*^*CreERT2/+*^ and *Wnt2*^*CreERT2/CreERT2*^ E17.5 heart and lung. (D) Proportion of different cell types in *Wnt2*^*CreERT2/+*^ and *Wnt2*^*CreERT2/CreERT2*^ embryonic heart and lung by scRNA-seq. (E and F) H&E images of (E) *Wnt2*^+/+^ and (F) *Wnt2*^*CreERT2/CreERT2*^ E17.5 lungs. (G and H) IHC for EMCN (G) to quantify vessel density of (G) *Wnt2*^+/+^ and (H) *Wnt2*^*CreERT2/CreERT2*^ E17.5 lungs. (I and J) IHC for ERG to quantify the endothelial cells in (I) *Wnt2*^+/+^ and (J) *Wnt2*^*CreERT2/CreERT2*^ E17.5 lungs. (K) Vascular density of lung between *Wnt2*^+/+^ and *Wnt2*^*CreERT2/CreERT2*^, *n* = 3 embryos. Data are represented as mean ± SEM. *p < 0.05; Student’s t test. (L) Percentage of ERG^+^ cells between *Wnt2*^+/+^ and *Wnt2*^*CreERT2/CreERT2*^, *n* = 3 embryos. Data are represented as mean ± SEM. ns, not significant; Student’s t test. (M and N) UMAPs embedded by expression of (M) *Wnt2b* and (N) *Wnt2* expression between *Wnt2*^*CreERT2/+*^ (left) and *Wnt2*^*CreERT2/CreERT2*^ (right). Scale bars: (E)–(H) 100 μm; (I) and (J) 50 μm.

Analyses of scRNA-seq data are useful for not only generating cell atlases but also for defining changes in cell composition and differential gene expression in loss-of-function studies. Previous studies characterizing loss of WNT2 in heart and lung development identified muscular hypoplasia as the predominant phenotype.^3,6,7,10,56^ However, we asked whether scRNA-seq could identify additional deficits with loss of WNT2. The *Wnt2*^*CreERT2*^ allele has an inducible Cre recombinase transgene knocked into the *Wnt2* locus, creating a haploinsufficient allele. Thus, we performed crosses to generate heterozygous (control) and homozygous (mutant) *Wnt2* mice and lineage traced from E8.5 to E17.5 to assess changes in *Wnt2*^+^ CPP progeny. We performed scRNA-seq on captured cells and generated UMAPs colored by cell type. Qualitatively, there was a reduction in CMs and endothelium (Figures 7A and 7B). Using quantitative cell number and proportion analysis, we noted similar cell reductions but concomitant with a gain in *Wnt2*^+^ mesoderm, smooth muscle, and pericytes (Figures 7C and 7D). To confirm CM reduction, we performed hematoxylin and eosin (H&E) staining of heart tissue sections of wild-type (WT), heterozygous, and homozygous mutant embryos at E17.5 (Figures S7A–S7C). We measured ventricle thickness in four areas of the heart, including RV free wall, RV apex, LV free wall, and LV apex and confirmed a reduction in the thickness of mutant ventricles (Figures S7D and S7E). Similarly, we qualitatively examined lung tissue sections after H&E staining and observed saccular simplification in *Wnt2* mutant lungs at E17.5 (Figures 7E and 7F). In addition, we performed fluorescent IHC with EMCN to identify capillaries and veins and with ERG to stain nuclei of all ECs and quantified vessel density and total EC number, respectively (Figures 7G–7J). Although quantification demonstrated a modest reduction in the density of capillary endothelium, there were no differences in total EC numbers in the *Wnt2* mutant lungs (Figures 7K and 7L).^40^ Similarly, we noted no difference in TAGLN^+^ VSM cell numbers per cross-sectional area of a vessel, independent of vessel size (Figures S7F–S7H). This mild phenotype was not a result of compensatory expression of *Wnt2b* (Figure 7M), but there appeared to be incomplete deletion of *Wnt2* as assessed by UMAPs colored by the expression of *Wnt2* (Figure 7N). Despite a modest endothelial phenotype, there were clear differences in differential gene expression analysis on ECs in the scRNA-seq data from control and mutant lungs (Tables S1 and S2). Multiple genes important for endothelial growth and maturation, including *Foxf1*, *Atf3*, *Egr1*, and *Junb*, were significantly downregulated in our scRNA-seq data comparing *Wnt2* mutant lungs relative to heterozygous controls (Figure S7I).^57–61^

## DISCUSSION

Using combined *in vivo* lineage tracing with scRNA-seq, our study has provided a comprehensive developmental cell atlas of differentiating cardiopulmonary mesodermal progenitor cells. We have identified a number of previously underappreciated progeny of CPPs in the heart and lung, in addition to providing clarity to specific subpopulations of the lung mesoderm.^6^ Importantly, we have not only captured some of the earliest molecular and cellular events described in the developing lung mesoderm but also refined cardiac mesodermal subpopulations and their molecular programs guiding maturation. This study highlights the importance of CPP specification and is one of the first studies to combine *in vivo* lineage tracing and scRNA-seq in the developing lung and heart. Moreover, these data will help highlight early and late molecular mechanisms driving cardiopulmonary mesodermal progenitor cell fate.

### *Wnt2*^+^ CPPs in cardiac mesodermal cell specification

These experiments add to the growing single-cell transcriptomic and epigenomic data instructing cardiac cell fate. A large majority of these data assessed the early stages of cardiac development, providing key information guiding early CM development.^23–33^ Here, we present data from *Wnt2*^+^ CPPs across prenatal development that will assist in the identification of the determinants of late mesodermal lineage allocation and CM identity and maturation.^3,6–8,32^ Additionally, we used RNA FISH and transcriptome analysis to spatially segregate SA, SV, and PV CMs, providing spatial information that may help clarify cell-specific determinants of posterior SHF progenitor cells.^62^ Interestingly, we did not observe *Wnt2*^+^ CPPs as a contributor to cardiac fibroblasts, pericytes, or endocardium. Rather, they are a newly identified source for the cellular composition of the atrioventricular valves, epicardium, and pericardial sac, including pericardium and pericardial interstitial cells. These findings are consistent with previous studies identifying *Isl1*^+^ cardiac mesodermal progenitor cells as a source for some cells of not only the proepicardium but also subsets of LV CMs seen with our data.^4,63,64^ In addition, our RNA FISH data showing *Wnt2* and *Wt1* colocalizing in the area of the SHF and proepicardial organ provide additional support for *Wnt2*^+^ CPPs contributing to progenitors of the epicardium, pericardium, and mesothelium. These findings are also consistent with progenitors of the proepicardial organ arising from the lateral plate mesoderm.^42,43^

### *Wnt2*^+^ CPPs in lung mesodermal cell specification

In this study, we have expanded on previously reported mesodermal lineages derived from CPPs. We confirmed not only known CPP-derived vascular and airway SMCs, pericytes, and *Wnt2*^+^ mesodermal cells but also novel derivation of the distal capillary endothelium and mesothelium of the lung. Previously, data indicated that *Wnt2*^+^ CPPs gave rise to the proximal pulmonary endothelium exclusive of the distal capillary endothelial bed. We hypothesized that the increased representation of CPP-derived cell types, including the capillary ECs in our study, may be a result of varying recombination efficiencies using different reporter constructs in the Rosa26 locus (R26R-CAG-mTmG^6^ vs. R26R-EYFP in our study).^65–67^ Indeed, our data rigorously demonstrate a decrease in recombination efficiency in combination with the *Wnt2*^*CreERT2*^ allele. Nevertheless, these data provide information on the earliest stages of lung mesodermal development at the single-cell level, providing a map of early specification.

### CPPs as a heterogeneous vs. homogeneous cardiopulmonary progenitor pool

*Wnt2*^+^ CPPs give rise to both cardiac and lung mesodermal lineages. Whether *Wnt2*^+^ CPPs represent a common pool of early mesodermal progenitors capable of producing both organ lineages is unclear. Using a combination of multiplex RNA FISH and computational integration of an elegant dataset of scRNA-seq work on dissected anterior foregut mesoderm and endoderm at E8.5 and E9.5,^8^ our results suggest that CPPs are a heterogeneous population of cell types that express *Wnt2*. However, original work using clonal lineage tracing identified that a portion of the individual CPP clones contained both cardiac and lung lineages.^6^ These data suggest that within *Wnt2*^+^ mesodermal cells, there may still be a small population of progenitor cells with the ability to differentiate into both cardiac and lung lineages. Alternatively, *Wnt2*^+^ CPPs at E8.5 may mark the anterior lateral plate mesoderm that gives rise to both SHF cardiac mesoderm and splanchnic mesoderm that soon separate into heart and lung lineages. Recent technology using barcode labeling technologies may help answer this question in future studies.

### WNT2 promotes CM development and lung maturation

Loss of WNT2 globally led to hypoplasia of the ventricular myocardium of the heart with a mild effect on vascular development. Previously, global deletion of WNT2 led to primarily a muscle phenotype with hypoplasia of the ventricular and pulmonary venous myocardium along with a reduction in smooth muscle in the lung.^7,10^ We did not observe all of these defects associated with loss of WNT2, but our results may reflect incomplete deletion of WNT2, as noted in our scRNA-seq data. Moreover, this *Wnt2* loss-of-function allele is slightly different from that previously published.^7,68,69^ While our data indicated that there is a loss of endothelium in the lung by proportional analysis of scRNA-seq data and vascular density measurements, the total number of ECs did not differ between control and mutant embryonic lungs. This may reflect the immaturity of the developing saccule and capillary bed as it remodels rather than a decrease in EC numbers. Nevertheless, phenotypes generated by scRNA-seq should be complemented by rigorous morphological and molecular assessments.

### Limitations of the study

In these studies, it is unlikely that we were able to label all of the *Wnt2*^+^ CPP-derived cells. With the use of spatiotemporal cell fate mapping during development, recombination efficiency is subject to the strength of the promoter driving Cre recombinase in addition to its efficiency activating the reporter. Hence, the system may label a smaller portion of CPP-derived cells than is truly present. However, we cannot neglect the contribution of other early mesodermal progenitor populations not labeled by *Wnt2* expression. The use of a constitutive *Wnt2*^*Cre*^ or additional Cre recombinases driven by alternative CPP-specific gene promoters (Isl1, Gli1, Osr1) may improve labeling efficiency and enable a more comprehensive characterization of the extent of contribution of CPPs to cardiopulmonary cell fate.

## STAR★METHODS

### EXPERIMENTAL MODEL AND STUDY PARTICIPANT DETAILS

#### Animal models

The *Wnt2*^*CreERT2*^, *R26R*^*mTmG*^(JAX, 007576) and *R26R*^*EYFP*^ (JAX, 006148) mice and genotyping information were previously described.^6,52,79^ Timed matings were set up counting day of plug as 0.5. The developmental ages of mouse embryos used in each experiment are shown in figures, figure legends, and results text. All mice were maintained on a CD-1 and C57BL6/J mixed background. For all experiments, both male and female mouse embryos were used. All animal studies were performed in adherence to the guidelines of the Children’s Hospital of Philadelphia Institutional Animal Care and Use Committee.

### METHOD DETAILS

#### Lineage tracing

Tamoxifen (Sigma-Aldrich) was dissolved in a mixture of 100% ethanol and corn oil (10% final volume of ethanol and 90% final volume of corn oil). To achieve successful recombination, pregnant females were injected with tamoxifen intraperitoneally at E8.5 at a concentration of 200 mg/gm of mouse.

#### Isolation and preparation of cells for scRNA-seq

Single-cell suspensions were made from tissue dissected from the thoracoabdominal area of two litters of whole embryos at E10.5, combined heart and lung dissected out from four embryos at E12.5, and four separated hearts that included the pulmonary vein and artery up to the hilum of the lung at E17.5, and four separated lungs containing only intrapulmonary tissue at E17.5. EYFP+ cells were isolated via FACS. Cells were processed for downstream analysis using the 10X Genomics 3^′^ gene expression scRNA-seq assay v3 protocol as previously described.^40^

#### Fluorescence-activated cell sorting (FACS)

FACS-based isolation of cells was performed as previously described.^40,53–55^ To isolate EYFP+ cells for scRNA-seq, single-cell suspensions were sorted using a FACSJazz flow sorter (BD Biosciences). For comparing the recombination efficiency of *R26R*^*mTmG*^ and *R26R*^*EYFP*^ reporter alleles, single cell suspensions were stained with DRAQ7 (Biolegend) to detect live/dead cells. Detection of cells, gating and use of filters including mGFP and YFP separate filters were performed using an Aurora A spectral analyzer (Cytek Biosciences).

#### Analysis of data from scRNA-seq

For each sequencing library, reads in FASTQ format were aligned to the NCBI RefSeq GRCm39 (GCF_000001635.27) genome and were quantified at the gene level by counting unique molecular identifiers (UMIs) using STAR-solo and the parameters *soloUMIdedup=“1MM_CR”*, *soloUMIfiltering=“MultiGeneUMI_CR”*, and *soloCellFilter=“EmptyDrops_CR”*.^70,71^ Ambient RNA-corrected counts matrices were generated using the scvi-tools implementation of the scAR algorithm.^72^ Gene counts matrices were subsequently processed using the scanpy and anndata python libraries.^73,74^

Cells with less than 1000 unique genes detected/cell, more than5000 unique genes detected/cell, or with more than 15% of unique reads attributed to mitochondrial genes were removed from the integrated dataset. Genes detected in fewer than 5 cells were additionally filtered from the data. Putative cell doublets were identified and removed using the scvi-tools implementation of the SOLO doublet identification model.^75^

Gene expression was calculated from counts matrices by normalizing to counts-per-million (CPM) using the *sc.pp.normalize_total* function with *target_sum=1e6* and then log-transforming these CPM values to ln(CPM +1) using the *sc.pp.log1p* function. The top 2000 highly variable genes were identified using the *sc.pp.highly_variable_genes* function, with *flavor=“seurat_v3”* and *n_top_genes=2000*. Principal component analysis (PCA) was performed using the *sc.tl.pca* function with *svd_solver= “arpack”,* and this PCA was subsequently batch-corrected using the pytorch implementation of the harmony algorithm.^76^ A cell-cell neighborhood was generated using the *sc.pp.neighbors function*, with n_neighbors=int(0.25 * np.sqrt(adata.n_obs)) and used to generate a 2-dimensional UMAP using the *sc.tl.umap* function with *init_pos=“spectral”*.^80^ Cell clustering was performed on the cell-cell network using the leiden algorithm with the *sc.tl.leiden* function with *resolution=1*.^81^ Cell compartment/cell type labels were manually assigned on a per-cell-cluster (leiden cluster) basis, based on expression of known marker genes.

Pseudotime trajectory inference was performed using *palantir* with CPPs from E10.5 selected as the root of the trajectory all other settings left to defaults.^77^ Inferred branches were assigned based on maximal branch probabilities. Gene expression trends were fitted to gene expression values associated with cells belonging to each of the identified trajectory branches using pyGAM.^78^ CPPs were included in each branch for the purposes of gene expression trend fitting.

Differential gene expression testing was performed using the *scanpy sc.tl.rank_genes_groups* function with *test=‘t test_overestim_var’*. Results were filtered using the *sc.tl.filter_rank_genes_groups* function to include only those genes expressed at greater than zero levels in more than 50% of cells in the tested group and less than 35% of cells in all other groups.

Integration of the scRNA-seq data generated in this study with the scRNA-seq data generated by Han et al. was performed by merging the raw counts and metadata matrices provided by the original authors (GSE136689) with the raw counts and metdata matrices generated in this scRNA-seq study.^8^ Genes not expressed/detected in both datasets were removed before subjecting this joint dataset to the same QC filtering and downstream processing procedures outlined above. Cell type, embryo stage, and tissue compartment labels were carried over from each original dataset’s annotations.

#### Histology, immunohistochemistry and RNA *in situ* hybridization

Embryonic lungs and hearts were harvested at E10.5, E12.5, and E17.5, and adult lungs and hearts were harvested at P42. For knock out studies, both hearts and lungs were harvested from the wild type, heterozygous *Wnt2*^*CreERT2/+*^, and homozygous *Wnt2*^*CreERT2/CreERT2*^ embryos at E17.5. For comparing the recombination efficiency of *R26R*^*mTmG*^ and *R26R*^*EYFP*^ reporter alleles, both hearts and lungs were harvested from the wild type, *Wnt2*^*CreERT2/+*^; *R26R*^*EYFP/+*^, *Wnt2*^*CreERT2/+*^; *R26R*^*mTmG/+*^, and *Wnt2*^*CreERT2/+*^; *R26R*^*EYFP/mTmG*^ embryos. After harvest, the right lobes were used to generate single-cell suspensions for FACS analysis as described above, and the left lobes were fixed using 2% paraformaldehyde (PFA) in PBS (ThermoScientific) O/N at 4°C. Samples were then washed, dehydrated, and embedded in paraffin. Using a microtome (Leica), we generated 6-μm transverse and sagittal sections for E10.5 and E12.5. Heart and lungs were embedded and sectioned separately at E17.5 and P42. Sections were deparaffinized and rehydrated, and every 5^th^ section was exposed to Hematoxylin and Eosin (Sigma-Aldrich) staining to visualize the gross morphological changes.

For fluorescent immunohistochemistry, subsequent sections from above were incubated with antigen retrieval (Reveal Decloaker, Biocare Medical) to expose antigens. These sections were then quenched of peroxidases activity using 3% H_2_O_2_ for 15 min, blocked with 5% donkey serum (Sigma-Aldrich) for 1 h and then incubated with primary antibodies (Table S3) O/N at 4°C. Subsequently, the relevant protein staining was visualized using Alexa Fluor secondary antibodies (Invitrogen) for 1 h at the dilution of 1:200 in 0.1% PBS-T. Finally, slides were incubated in DAPI (Invitrogen, D1306) for 10 min and sealed using VECTASHIELD antifade mounting medium (Vector Laboratories) before confocal microscopy imaging.

On further sections, RNA fluorescence *in situ* hybridization (FISH) was performed with RNAscope multiplex fluorescent V2 assay kit (323100, Advanced Cell Diagnostics (ACDbio), Newark, CA), as previously described.^40^ RNAscope 3-plex negative control probe and mouse specific 3-plex control probe were used for control sections. Briefly, the sections were deparaffinized, hydrated and probe targets were retrieved using 1x target retrieval for 10 min at 98°C–102°C. Sections were further incubated in protease plus for 15 min and following hybridization and channel development, the probes were imaged at relevant channels using Opal fluorophore reagents (Opal 520 FP1487001, Opal 540 FP1494001, Opal 570 FP1488001 or Opal 650 FP1496001, Akoya Biosciences). Finally, slides were incubated in DAPI for 10 min and sealed using Prolong gold antifade mount (Invitrogen).

#### Whole mount fluorescent IHC and RNA FISH

Whole mount immunofluorescence staining was performed as previously described.^40^

Briefly, after overnight fixation in 2% paraformaldehyde in PBS, the lung tissue was washed with cold PBS and then embedded in 4% low melt agarose (ThermoFisher). The embedded lungs were sectioned at 150 μm thickness via a Vibratome (Leica). Sections were then incubated with primary antibody in 5% donkey serum and 0.5% PBS-T for 4 h at room temperature and then overnight at 4°C. Subsequently, tissue sections were incubated with secondary antibody in 5% donkey serum and 0.5% PBS-T for 1 h at room temperature and then overnight at 4°C. After washes, the sections were fixed in 2% PFA for 30 min at room temperature and then cleared with Scale A2 before confocal microscopy imaging as described previously.^38^

Whole mount RNA *in situ* hybridization was performed on E8.5 embryos using a modified previously established protocol.^82^ Embryos were carefully harvested, fixed O/N in 2% paraformaldehyde at 4°C. Embryos were then washed with PBT for 5 min each, 3 times before dehydrating with graded methanol/PBT (MeOH/PBT) mixture for 5 min each. Samples were rehydrated again in the reverse gradation of MeOH/PBT for 5 min each and were washed again in PBT 3 times for 5 min each. Samples were then treated with Protease III for 10 min at 40°C on a shaker to facilitate digestion and then probes were hybridized for 12–16 h at 40°C. Each subsequent channels were developed separately with HRP-C1/C2/C3, Opal fluorophore reagents (see above, RNA FISH protocol) and finally, sections were incubated with HRP blocker for 40 min at 40°C between each channel development. Embryos were then incubated with DAPI for 15 min and then fixed with 2% paraformaldehyde. Finally, embryos were cleared with CUBIC (RIA) reagent and sealed using prolong gold antifade mountant media before imaging.^83^

### QUANTIFICATION AND STATISTICAL ANALYSIS

#### Quantification of reporter recombination efficiency

Cells were prepared as described above for FACS analysis in the CHOP Flow Core. A minimum of 50,000 events were collected. Data were analyzed using FlowJo software (version 10).

To compare the percentage of capillary endothelial cells within EYFP+ and mGFP+ cell populations, we performed whole mount immunofluorescence staining of EMCN on E17.5 precision-cut lung slices (PCLS) as described above. Images were captured using a Leica SP8 confocal microscope with a 40× objective at five random reporter-positive cell clusters across the PCLS for each sample. The size of z stack of was set to 150 μm. These images were then processed with ImageJ (Fiji), and cells with colocalized signals were manually counted with the multi-point counting tool. We counted the EMCN+, EYFP+ cells and EMCN+, mGFP+ cells and normalized it over all EMCN+ cells in each image.

#### Quantification of vascular density

Changes in vascular density were quantified at E17.5 lungs using a previously described approach.^40^ IHC for EMCN antibody was performed on sections as noted above. Vessel density was measured on whole lung tile scans using 203 magnification confocal z-stacks using the IMARIS 9.7.2 software. Whole lung tile scans were obtained from control *Wnt2*^+*/CreERT2*^ and homozygous null *Wnt2*^*CreERT2/CreERT2*^ embryos. From these images, corresponding DAPI channel was used to obtain the 3D reconstruction of whole volume of the tissue, where the total tissue volume was acquired using the volume statistics function in IMARIS. Using EMCN (capillary and veins) staining for volume of lung vasculature, we measured the vessel density of the tissue by normalizing the vessel volume with total lung volume obtained from the DAPI channel.

#### Quantification of pulmonary endothelial cell proportion

To measure the changes in endothelial cell proportion after deletion of WNT2, immunofluorescence staining for ERG and DAPI was performed on paraffin-embedded E17.5 lung sectioned as described above. Whole lung tile scans were obtained using a Leica SP8 confocal microscope with a 20× objective. Using the spot detection and colocalization analysis tool in IMARIS 9.7.2 software, we automatically quantified the number of lung cells in the section, marked by DAPI, and the number of pulmonary endothelial cells, marked by ERG and DAPI. The percentage of pulmonary endothelial cells was calculated by dividing the number of ERG+, DAPI+ cells by the number of DAPI+ cells.

#### Quantification of vascular smooth muscle cells

To measure the TAGLN+ cells surrounding the proximal vessels, IHC staining for TAGLN and DAPI was performed on paraffin-embedded E17.5 lung sectioned as described above. Images were captured using a Leica SP8 confocal microscope with a 40× objective at five random vessels across the section for each sample. These images were then processed in ImageJ, and cells with colocalized signals were manually counted with the multi-point counting tool. We counted the number of TAGLN+ DAPI cells surrounding the vessel and normalized it over the number of DAPI cells in the corresponding vessel.

#### Quantification of ventricular hypoplasia

To measure the ventricular hypoplasia, H&E staining on E17.5 heart sections was performed. Whole heart tile scans were captured using a Leica DMI8 Thunder microscope with a 20× objective. These images were then processed in ImageJ. Four regions of interest (ROIs’s) in ventricular regions of heart were identified, namely, left ventricle (LV) free-wall, LV apex, right ventricle (RV) apex and RV free-wall. In each of these regions, 10 measurements were taken, and the mean thickness was calculated.

#### Statistical analysis

Unpaired, two-tailed Students t-tests were applied to detect significant differences in all quantification assays. For all the tests, the significance level was set at α = 0.05. All statistical tests were performed using GraphPad Prism 9. Specific *n* values are listed in the figure legend. In all graphs, each individual data point represents an animal or litter. * represents *p* < 0.05.

## Supplementary Material

1

2

## Figures and Tables

**Table:** KEY RESOURCES TABLE

REAGENT or RESOURCE	SOURCE	IDENTIFIER
Antibodies		
Rabbit polyclonal Anti-ESRRG antibody	Dr. Ronald M. Evans	Wang et al.,^46^ Pei et al.^45^
Rabbit polyclonal Anti-SOX6 antibody	Abcam	ab30455; RRID: AB_1143033
Goat polyclonal Anti-GFP antibody	Abcam	ab6673; RRID: AB_305643
Chicken polyclonal Anti-GFP antibody	Aves Labs	GFP-1020; RRID: AB_10000240
Mouse monoclonal Anti-ACTA2 antibody	Sigma-Aldrich	A5228; RRID: AB_262054
Goat polyclonal Anti-TAGLN antibody	Abcam	ab10135; RRID: AB_2255631
Rabbit polyclonal Anti-von Willebrand Factor antibody	Sigma-Aldrich	F3520; RRID: AB_259543
Goat polyclonal Anti-Endomucin antibody	R&D Systems	AF4666; RRID: AB_2100035
Rat monoclonal Anti-Endomucin antibody	Invitrogen	14-5851-85; RRID: AB_891531
Mouse monoclonal Anti-ERG antibody	Abcam	ab214341; RRID: AB_3073833
Rabbit monoclonal Anti-Wilms Tumor Protein antibody	Abcam	ab89901; RRID: AB_2043201
Mouse monoclonal Anti-Troponin T (Cardiac Isoform Ab-1) antibody	Epredia	MS-295-P1; RRID: AB_61808
Rat monoclonal Anti-CD31 (PECAM-1) antibody	HistoBio Tec	DIA-310; RRID: AB_2631039
Goat polyclonal Anti-PDGF Receptor beta antibody	R&D Systems	AF1042; RRID: AB_2162633
Rabbit monoclonal Anti-PDGF Receptor alpha antibody	Cell Signaling	3174S; RRID: AB_2162345
Donkey anti-Goat IgG (H + L) Highly Cross-Adsorbed Secondary Antibody, Alexa Fluor^™^ Plus 488	Invitrogen	A32814; RRID: AB_2866497
Donkey anti-Chicken IgG (H + L) Highly Cross-Adsorbed Secondary Antibody, Alexa Fluor^™^ 488	Invitrogen	A78948; RRID: AB_2921070
Donkey anti-Rabbit IgG (H + L) Highly Cross-Adsorbed Secondary Antibody, Alexa Fluor^™^ 488	Invitrogen	A21206; RRID: AB_2535792
Donkey anti-Mouse IgG (H + L) Highly Cross-Adsorbed Secondary Antibody, Alexa Fluor^™^ 555	Invitrogen	A31570; RRID: AB_2536180
Donkey anti-Rabbit IgG (H + L) Highly Cross-Adsorbed Secondary Antibody, Alexa Fluor^™^ 568	Invitrogen	A10042; RRID: AB_2534017
Donkey anti-Mouse IgG (H + L) Highly Cross-Adsorbed Secondary Antibody, Alexa Fluor^™^ 647	Invitrogen	A31571; RRID: AB_162542
Donkey anti-Goat IgG (H + L) Cross-Adsorbed Secondary Antibody, Alexa Fluor^™^ 647	Invitrogen	A21447; RRID: AB_2535864
Donkey anti-Rabbit IgG (H + L) Cross-Adsorbed Secondary Antibody, Alexa Fluor^™^ 647	Invitrogen	A31573; RRID: AB_2536183
Donkey Anti-Rat IgG H&L (Alexa Fluor^®^ 647) preadsorbed	Abcam	ab150155; RRID: AB_2813835
Chemicals, peptides, and recombinant proteins		
Tamoxifen	Sigma Aldrich	T5648, CAS 10540-29-1
Opal 520 Reagent Pack	Akoya Biosciences	FP1487001
Opal 540 Reagent Pack	Akoya Biosciences	FP1494001
Opal 570 Reagent Pack	Akoya Biosciences	FP1488001
Opal 650 Reagent Pack	Akoya Biosciences	FP1496001
Hematoxylin	Sigma Aldrich	H9627
Eosin	Sigma Aldrich	E4009
Reveal Decloaker	BioCare Medical	MSPP-RV 1000M
Donkey serum	Sigma Aldrich	D9663
Triton X-100	Sigma Aldrich	T8787
DAPI (4*’*,6-diamidino-2-phenylindole)	Invitrogen	D1306
VECTASHIELD antifade mounting medium	Vector Laboratories	H-1000–10
Prolong gold antifade mountant	Invitrogen	P36930
Low melt agarose	ThermoFisher	16520050
Tween 20	Sigma Aldrich	P1379
DRAQ7	Biolegend	424001
Critical commercial assays		
RNAscope^™^ multiplex fluorescent V2 assay kit	Advanced Cell Diagnostics	323100
Chromium single cell 3’ chemistry kit v3	10X Genomics	PN-1000075
Deposited data		
Raw and analyzed scRNA-seq data of *E*10.5, *E*12.5, and *E*17.5 *Wnt2*^*+/CreERT2*^; *R26R*^*EYFP/EYFP*^ lungs and hearts	GEO: GSE267719	N/A
Raw and analyzed scRNA-seq data of *E*17.5 *Wnt2*^*CreERT2/CreERT2*^ homozygous mutant and *Wnt2*^*+/CreERT2*^ heterozygous litter mate controls lungs and hearts	GEO: GSE267719	N/A
Codes	https://doi.org/10.5281/zenodo.14894765	N/A
Experimental models: Organisms/strains		
Mouse: B6.129X1-*Gt(ROSA)26Sor*^*tm1(EYFP)Cos*^/J	Jackson Laboratory	RRID:IMSR_JAX:006148
Mouse: *Wnt2*^*CreERT2*^	Peng et al.^6^	RRID: MGI:5544574
Mouse: B6.129(Cg)-*Gt(ROSA)26Sor*^*tm4(ACTB-tdTomato*^,^*-EGFP)Luo*^/J	Jackson Laboratory	RRID:IMSR_JAX:007576
Oligonucleotides		
Primer: EYFP, Wild Type Forward: AAGGGAGCTGCAGTGGAGTA	The Jackson Laboratory	Primer no. oIMR9020
Primer: EYFP, Wild Type Reverse: CCGAAAATCTGTGGGAAGTC	The Jackson Laboratory	Primer no. oIMR9021
Primer: EYFP, Mutant Forward: ACATGGTCCTGCTGGAGTTC	The Jackson Laboratory	Primer no. oIMR9102
Primer: EYFP, Mutant Reverse: GGCATTAAAGCAGCGTATCC	The Jackson Laboratory	Primer no. oIMR9103
Primer: MTMG, Wild Type Forward: GGCTTAAAGGCTAACCTGATGTG	The Jackson Laboratory	Primer no. oIMR0314
Primer: MTMG, Wild Type Reverse: GGAGCGGGAGAAATGGATATG	The Jackson Laboratory	Primer no. oIMR8546
Primer: MTMG, Mutant Forward: AATCCATCTTGTTCAATGGCCGATC	The Jackson Laboratory	Primer no. oIMR0092
Primer: MTMG, Mutant Reverse: CCGGATTGATGGTAGTGGTC	The Jackson Laboratory	Primer no. oIMR3449
Primer: Wnt2^CreERT2^, Common Forward: TGAGTCTCACCACTAGCCGCA	Peng et al.^6^	N/A
Primer: Wnt2^CreERT2^, Wild Type Reverse: ACTGGGAATCAGCCAGGGAGGGT	Peng et al.^6^	N/A
Primer: Wnt2^CreERT2^, Mutant Reverse: TCCAGGTATGCTCAGAAAACG	Peng et al.^6^	N/A
RNAScope Probe Mm-Wnt2	ACDBio	313601
RNAScope Probe Mm-Isl1-C3	ACDBio	451931-C3
RNAScope Probe Mm-Nkx2-5-C2	ACDBio	428241-C2
RNAScope Probe Mm-WT1-C2	ACDBio	432711-C2
RNAScope Probe Mm-Hoxb1-C3	ACDBio	541861-C3
RNAScope Probe Mm-Nrg1	ACDBio	418181
RNAScope Probe Mm-Gja5-C2	ACDBio	518041-C2
RNAScope Probe Mm-Hcn4-C2	ACDBio	421271-C2
RNAScope Probe Mm-Shox2-O1-C3	ACDBio	554291-C3
RNAScope Probe Mm-Abcc9	ACDBio	411371
RNAScope Probe Mm-Gpihbp1-C3	ACDBio	540631-C3
RNAScope Probe Mm-Pdgfrb-No-XHs	ACDBio	448421
RNAScope Probe Mm-Lgr6-C3	ACDBio	404961-C3
RNAScope Probe Mm-Fgfr4-C2	ACDBio	446381-C2
Software and algorithms		
ImageJ (Fiji)	National Institutes of Health (NIH)	https://imagej.nih.gov/ij/RRID: SCR_003070
GraphPad Prism 9 software	GraphPad Software, Inc.	https://www.graphpad.com/scientific-software/prism/RRID: SCR_002798
IMARIS 9.7.2	Oxford Instruments	https://imaris.oxinst.com/RRID: SCR_007370
FlowJo v10.10	BD Biosciences	https://www.flowjo.com/solutions/flowjo RRID: SCR_008520
STARsolo	Dobin et al.^70^ Benjamin et al.^71^	https://github.com/alexdobin/STAR
scAR	Sheng et al.^72^	https://github.com/Novartis/scAR
SCANPY	Wolf et al.^73^	https://github.com/theislab/Scanpy
Anndata	Isaac et al.^74^	github.com/theislab/anndata
Scvi-tools	Gayoso et al.^75^	https://github.com/scverse/scvi-tools
Harmony-pytorch	Korsunsky et al.^76^	https://github.com/lilab-bcb/harmony-pytorch
Palantir	Setty et al.^77^	https://github.com/dpeerlab/Palantir/
pyGAM	Serven et al.^78^	https://zenodo.org/records/1476122

## Data Availability

Further information and requests for resources and reagents should be directed to and will be fulfilled by the lead contact, David B. Frank (frankd@chop.edu). This study did not generate new materials. scRNA-seq data generated in this study have been deposited at the NIH Gene Expression Omnibus and made publicly available as of the date of publication. The accession number is listed in the key resources table. All code used in this study has been deposited at GitHub and is available to the public as of the date of publication. The link to the code is available in the key resources table. Any additional information required to reanalyze the data reported in this paper is available from the lead contact upon request.

## Highlights

- Combined lineage tracing and temporal scRNA-seq to study cardiopulmonary development
- *Wnt2*^+^ cardiopulmonary mesodermal progenitor cells (CPPs) are a heterogeneous progenitor pool
- CPPs give rise to diverse heart and lung lineages
- Loss of WNT2 results in lung capillary simplification and ventricular hypoplasia
