# Supplementary material for: A spatiotemporal cell atlas of cardiopulmonary progenitor cell allocation during development
Source: Cell Rep. Author manuscript; Available in PMC 2025 May 24. (PMC12103214; doi:10.1016/j.celrep.2025.115513)
Supplement: 1 [file NIHMS2076725-supplement-1.pdf]

Cell Reports, Volume *44*

Supplemental information

A spatiotemporal cell atlas of cardiopulmonary progenitor cell allocation during development

Hongbo Wen, Prashant Chandrasekaran, Annabelle Jin, Josh Pankin, MinQi Lu, Derek C. Liberti, Jarod A. Zepp, Rajan Jain, Edward E. Morrisey, Sylvia N. Michki, and David B. Frank

Figure S1

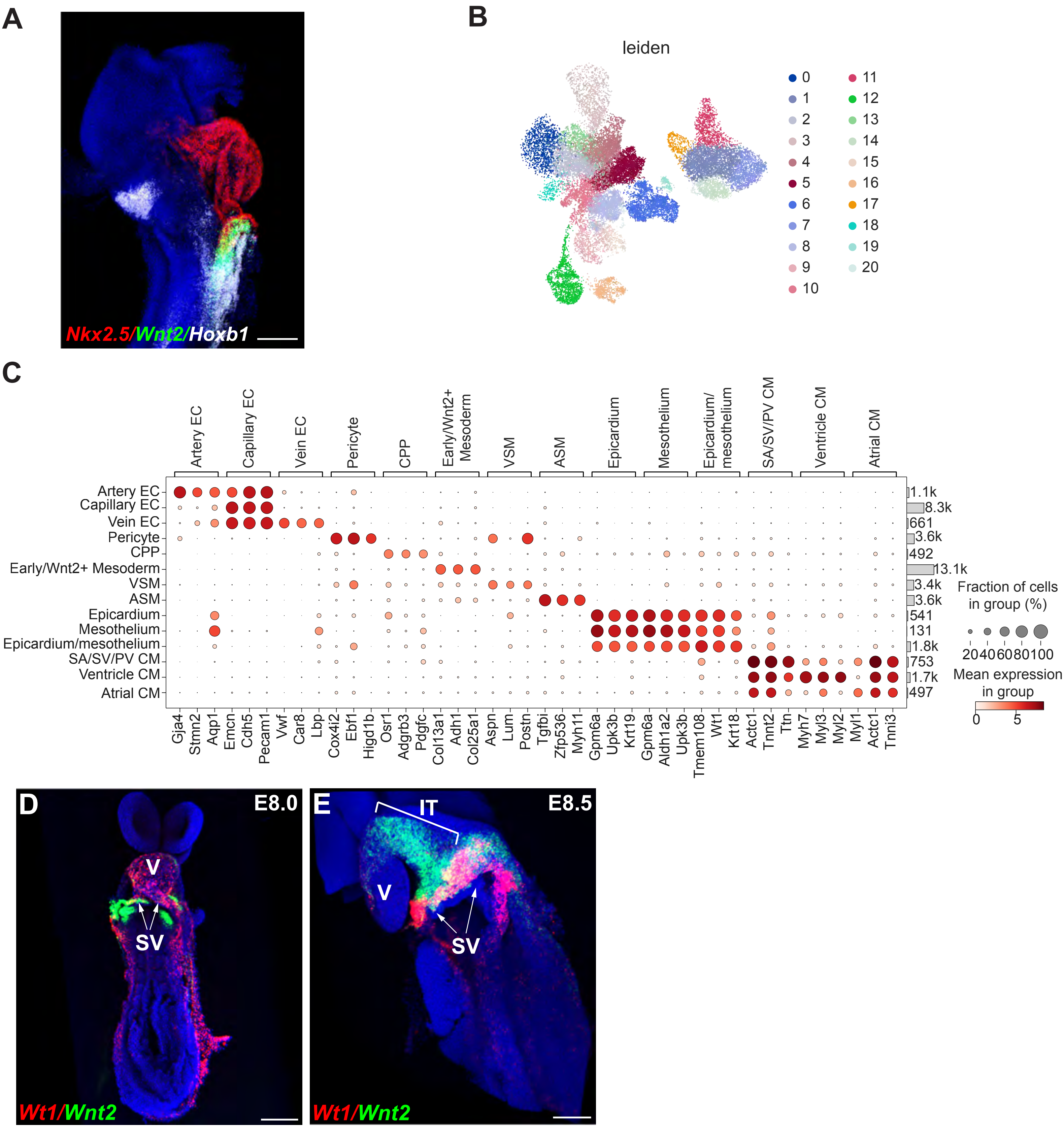
A
Nkx2.5/Wnt2/Hoxb1
B
leiden
0
1
2
3
4
5
6
7
8
9
10
11
12
13
14
15
16
17
18
19
20
C
Artery EC
Capillary EC
Vein EC
Pericyte
CPP
Early/Wnt2+ Mesoderm
VSM
ASM
Epicardium
Mesothelium
Epicardium/mesothelium
SA/SV/PV CM
Ventricle CM
Atrial CM
Gja4
Stmn2
Aqp1
Emcn
Cdh5
Pecam1
Vwf
Car8
Lbp
Cox4i2
Ebf1
Higd1b
Osr1
Adgrb3
Pdgfc
Col13a1
Adh1
Col25a1
Aspn
Lum
Postn
Tgfbi
Zfp536
Myh11
Gpm6a
Upk3b
Krt19
Gpm6a
Aldh1a2
Upk3b
Tmem108
Wt1
Krt18
Actc1
Tnnt2
Ttn
Myh7
Myl3
Myl2
Myl1
Actc1
Tnni3
1.1k
8.3k
661
3.6k
492
13.1k
3.4k
3.6k
541
131
1.8k
753
1.7k
497
Fraction of cells in group (%)
20
40
60
80
100
Mean expression in group
0
5
D
E8.0
V
SV
Wt1/Wnt2
E
E8.5
IT
V
SV
Wt1/Wnt2

Fig. S1. *Wnt2*+ CPPs spatially and temporally populate heart and lung cells, related to Figure 1. (A) Whole mount RNA FISH of E8.5 embryo for *Nkx2.5*/*Wnt2/Hoxb1*. (B) UMAP embedded with cells colored by Leiden clustering of EYFP+ cells from lung and heart at all time points. (C) Dot plot consisting of marker genes for cell types where dot size and color intensity indicates the cell proportion and gene expression, respectively, within the cluster. (D and E) Whole mount RNA FISH of E8.0 (D) and E8.5 (E) embryo for *Wnt2/Wt1*. V-ventricle, SV-sinus venosus, IT-inflow tract. Scale bar: 100 μm.

Figure S2

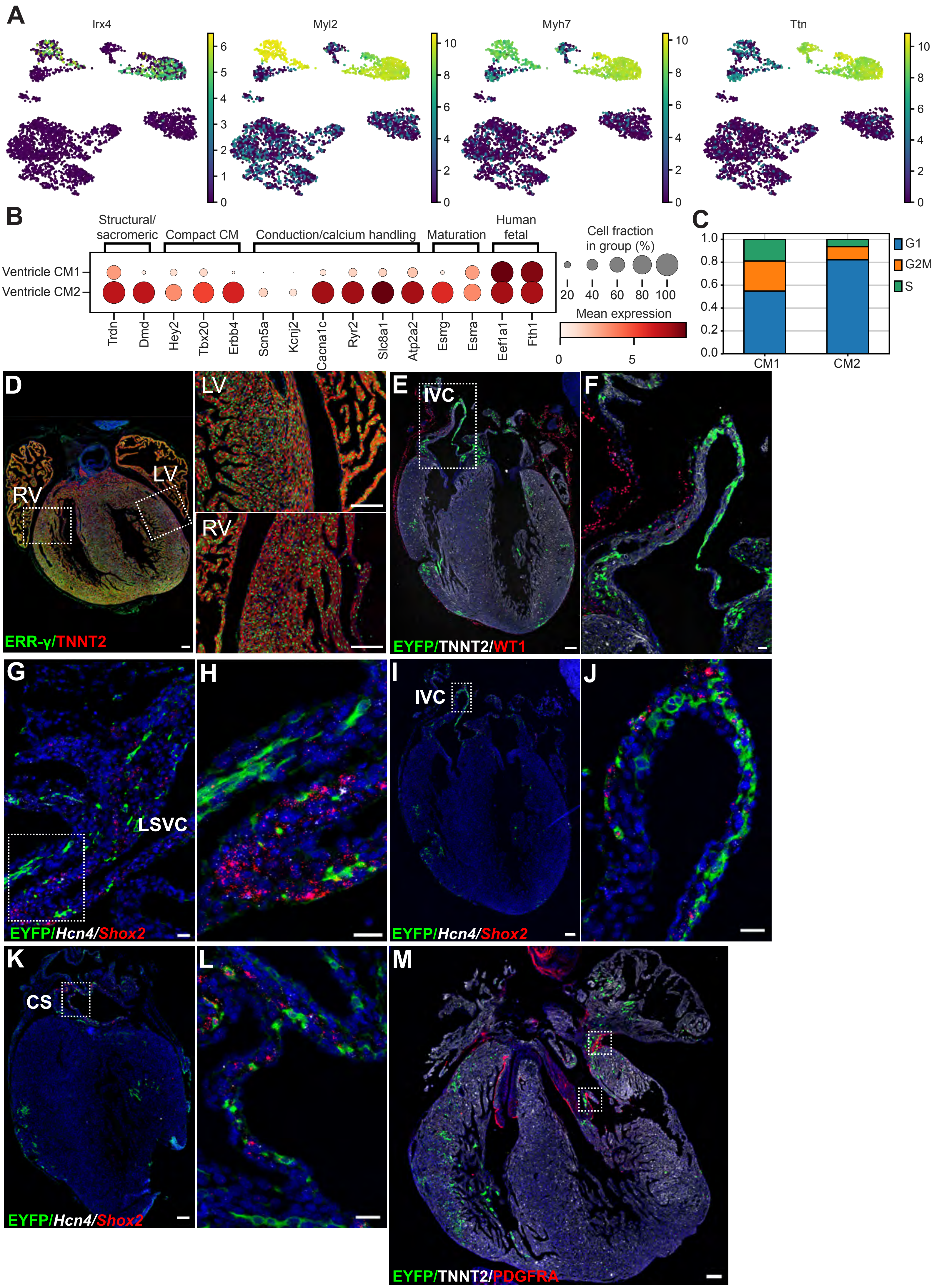
A
Irx4
Myl2
Myh7
Ttn
B
Structural/ sacromeric
Compact CM
Conduction/calcium handling
Maturation
Human fetal
Ventricle CM1
Ventricle CM2
Trdn
Dmd
Hey2
Tbx20
Erbb4
Scn5a
Kcnj2
Cacna1c
Ryr2
Slc8a1
Atp2a2
Esrrg
Esrra
Eef1a1
Fth1
Cell fraction in group (%)
20 40 60 80 100
Mean expression
0 5
C
G1
G2M
S
CM1
CM2
D
RV
LV
ERR-γ/TNNT2
E
IVC
EYFP/TNNT2/WT1
F
G
LSVC
EYFP/Hcn4/Shox2
H
I
IVC
EYFP/Hcn4/Shox2
J
K
CS
EYFP/Hcn4/Shox2
L
M
EYFP/TNNT2/PDGFRA

Fig. S2. *Wnt2*+ CPPs are specified into different cell lineages in the heart at E17.5, related to Figure 2. (A) UMAP embedded by the expression of *Irx4, Myl2, Myh7 and Ttn* at E17.5 heart. (B) Dot plot consisting of mature and fetal CM marker gene expression where dot size and color intensity indicate cell proportion and gene expression, respectively, within the identified cluster. (C) Proportion of cells in CM1 and CM2 clusters based on cell cycle phases. (D) IHC for TNNT and estrogen receptor gamma (ESRRG) with magnified areas of LV and RV in boxes. (E and F) IHC for TNNT2, WT1 and EYFP with (F) higher magnification of boxed area in (E) (IVC-inferior vena cava). (G-L) RNA FISH for *Hcn4/Shox2* with IHC for EYFP. (G and H) Image of left superior vena cava (LSVC) with (H) higher magnification of boxed are in (G). (I and J) Image of inferior vena cava (IVC) with (J) higher magnification of boxed area in (I). (K and L) Image of coronary sinus (CS) with (L) higher magnification of boxed area in (K). (M) IHC for TNNT2, PDGFRA and EYFP. Scale bar = 100 μm for D (left and right upper and lower panels), E, G, I, K and M, and 25 μm for F, H, J and L.

Figure S3

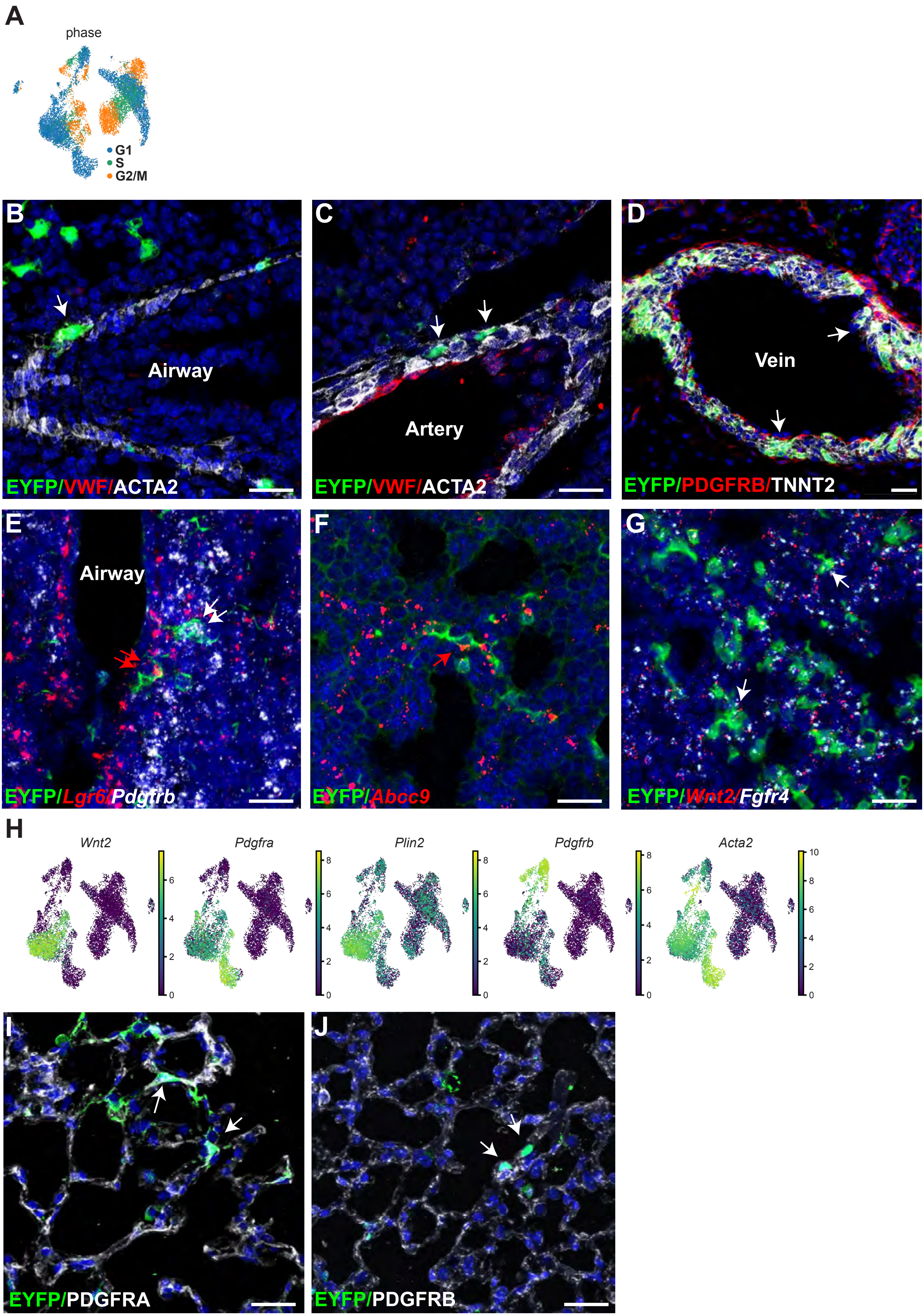
A
phase
G1
S
G2/M
B
Airway
EYFP/VWF/ACTA2
C
Artery
EYFP/VWF/ACTA2
D
Vein
EYFP/PDGFRB/TNNT2
E
Airway
EYFP/Lgr6/Pdgfrb
F
EYFP/Abcc9
G
EYFP/Wnt2/Fgfr4
H
Wnt2
Pdgfra
Plin2
Pdgfrb
Acta2
I
EYFP/PDGFRA
J
EYFP/PDGFRB

Fig. S3. *Wnt2*+ CPPs are specified into mesodermal lineages in the lung at E17.5 related to Figure 3. (A) UMAP embedding of lung cells colored by inferred cell cycle phases. (B and C) IHC for ACTA2, VWF, and EYFP on an airway (B) and artery (C). (D) IHC for PDGFRB, TNNT2, and EYFP on a vein. (E) RNA FISH on an airway for *Lgr6* and *Pdgfrb* and IHC for EYFP. (F) RNA FISH for pericytes in the alveolar region for *Abcc9* and IHC and EYFP. (G) RNA FISH in the alveolar region for *Wnt2/Fgfr4* and IHC for EYFP. (H) UMAPs colored by expression of *Wnt2*, *Pdgfra*, *Plin2*, *Pdgfrb*, and *Acta2*. (I and J) IHC for EYFP and (I) PDGFRA and (J) PDGFRB. Scale bar = 25 µm for B-J.

Figure S4

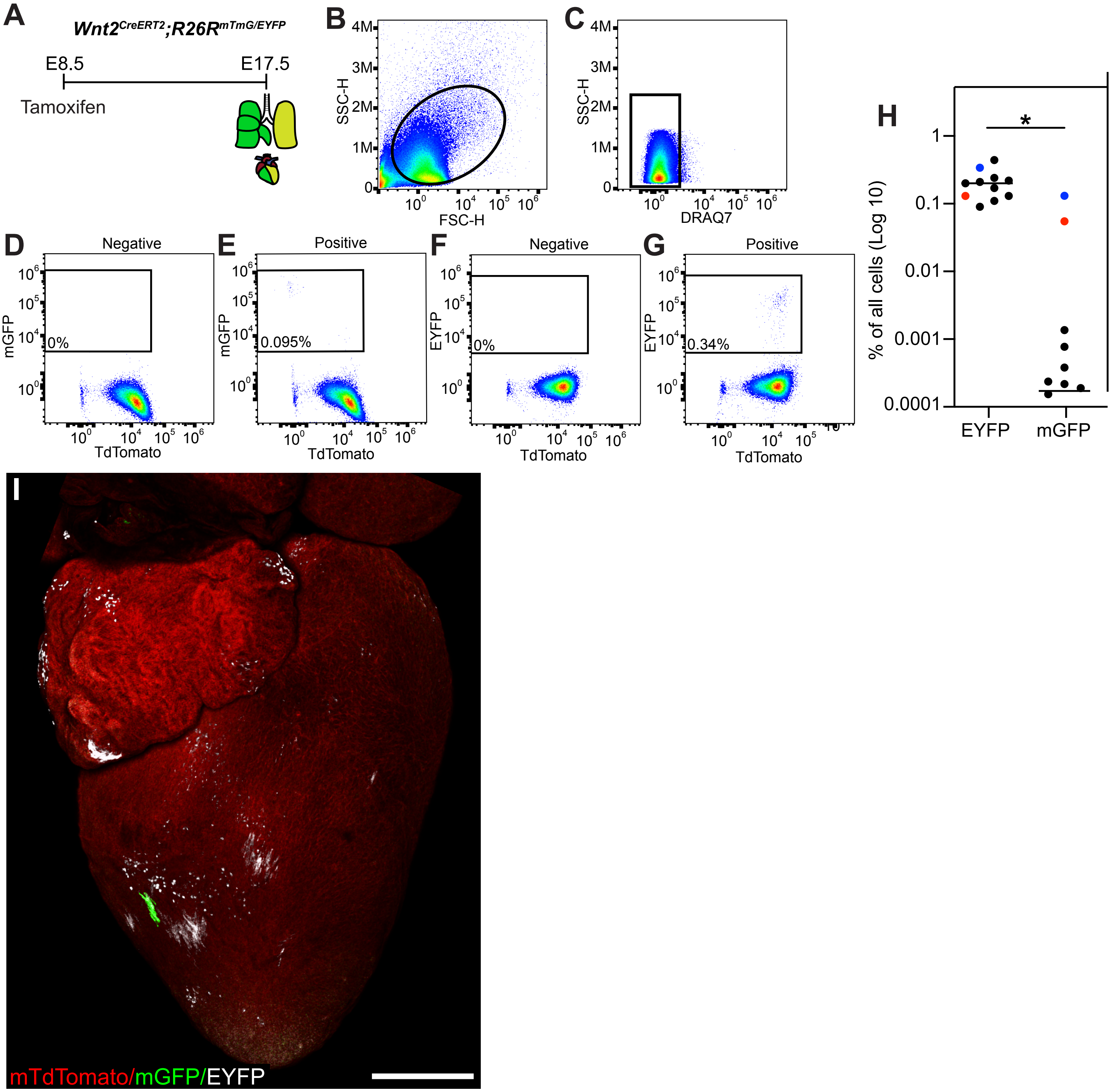

A
Wnt2CreERT2;R26RmTmG/EYFP
E8.5
E17.5
Tamoxifen
B
SSC-H
FSC-H
C
SSC-H
DRAQ7
D
Negative
mGFP
0%
TdTomato
E
Positive
mGFP
0.095%
TdTomato
F
Negative
EYFP
0%
TdTomato
G
Positive
EYFP
0.34%
TdTomato
H
% of all cells (Log 10)
*
EYFP
mGFP
I
mTdTomato/mGFP/EYFP

Fig. S4. Efficiency of different *Rosa26* reporters with *Wnt2*^{CreERT2}, related to Figure 4. (A) Tamoxifen administration and harvest of heart and lung at E17.5. (B-G) Representative FACS gating strategy for collection of (B) cells, (C) DRAQ7- live cells, (D) mGFP- cells, (E) mGFP+ cells, (F) EYFP- cells and (G) EYFP+ cells. (H) Percent of all recombined cells in EYFP+ and mGFP+ lung cells using a logarithmic scale. Red dots represent one sample embryo in a litter, and blue dots represent another sample embryos in a litter, n=11 embryos. *$p < 0.05$;Students t-test. (I) Whole mount imaging of native reporters, mTdtomato (red), mGFP (green) and EYFP (white), in E17.5 heart. Scale bar = 500 μm.

Figure S5

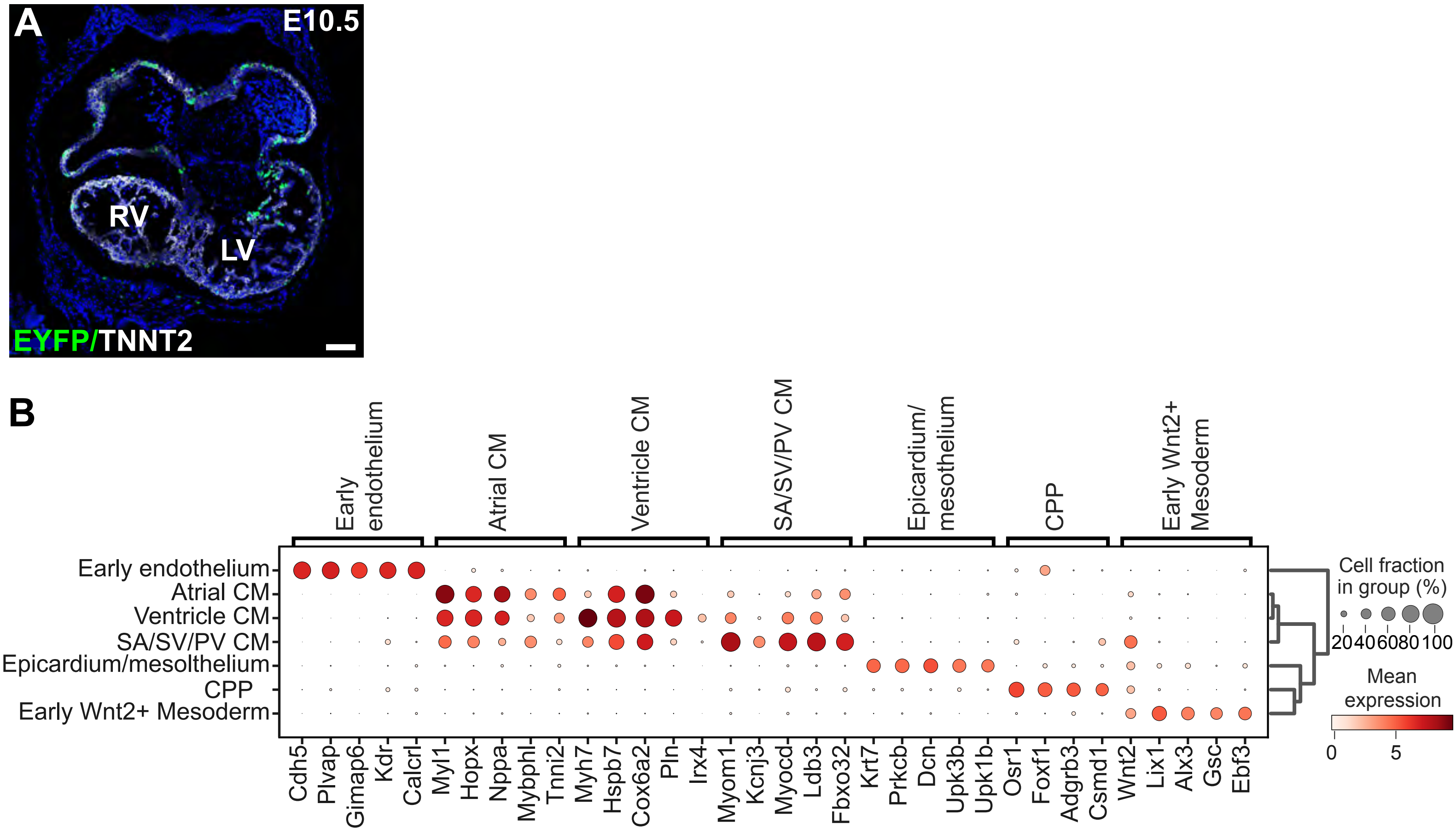
A
E10.5
RV
LV
EYFP/TNNT2
B
Early endothelium
Atrial CM
Ventricle CM
SA/SV/PV CM
Epicardium/ mesothelium
CPP
Early Wnt2+ Mesoderm
Early endothelium
Atrial CM
Ventricle CM
SA/SV/PV CM
Epicardium/mesolthelium
CPP
Early Wnt2+ Mesoderm
Cdh5 Plvap Gimap6 Kdr Calcrl Myl1 Hopx Nppa Mybphl Tnni2 Myh7 Hspb7 Cox6a2 Pln Irx4 Myom1 Kcnj3 Myocd Ldb3 Fbxo32 Krt7 Prkcb Dcn Upk3b Upk1b Osr1 Foxf1 Adgrb3 Csmd1 Wnt2 Lix1 Alx3 Gsc Ebf3
Cell fraction in group (%)
20 40 60 80 100
Mean expression
0 5

Fig. S5. Differential transcription factor expression in *Wnt2*+ CPP-derived progeny across development, related to Figure 5. (A) IHC for TNNT2 and EYFP in the heart at E10.5 (RV-right ventricle, LV-left ventricle). (B) Dot plot consisting of Leiden clustering-based marker genes for cell types where dot size and color intensity indicate cell proportion and gene expression, respectively, within the identified cluster. Scale bar: A = 100 μm.

Figure S6

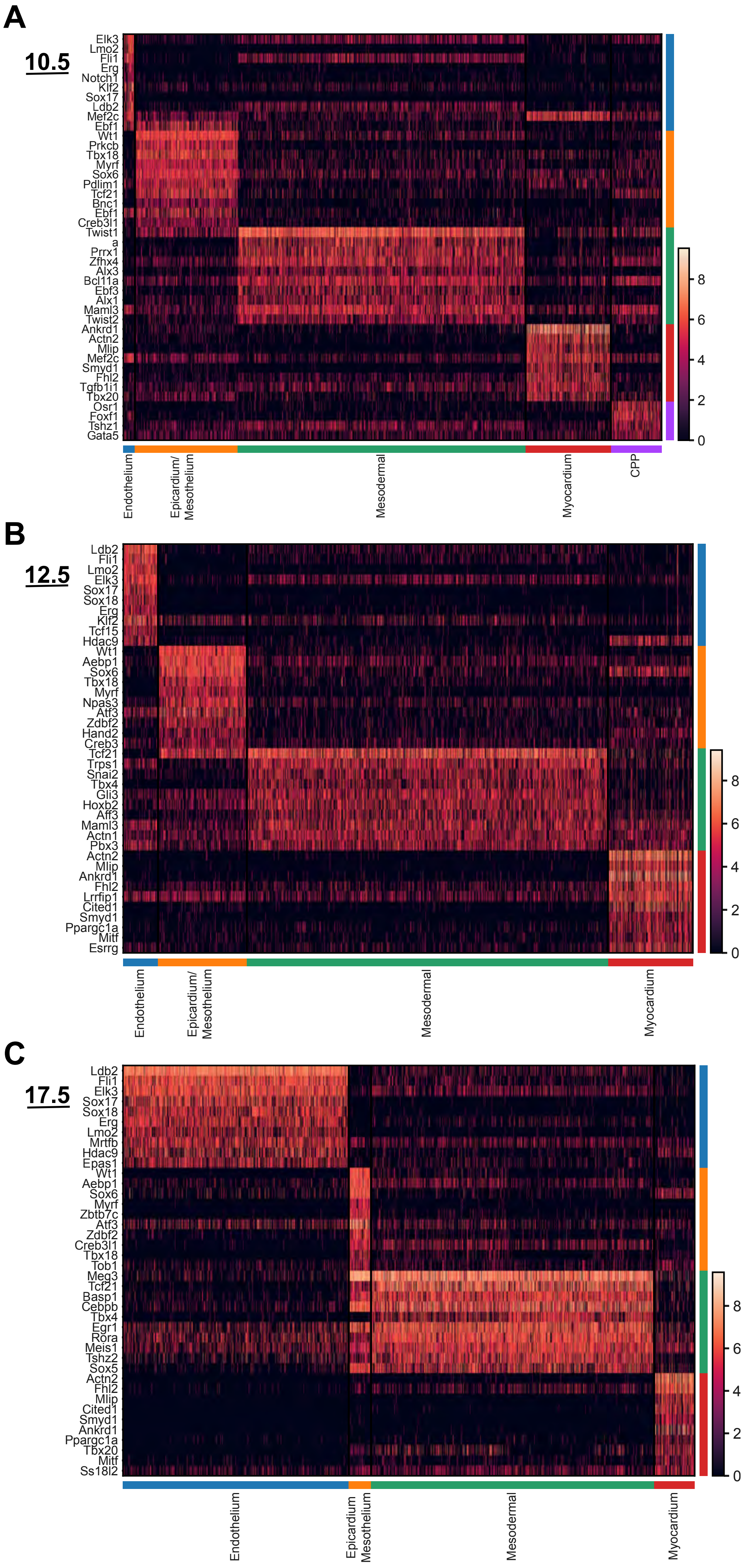
A
10.5
Elk3
Lmo2
Fli1
Erg
Notch1
Klf2
Sox17
Ldb2
Mef2c
Ebf1
Wt1
Prkcb
Tbx18
Myrf
Sox6
Pdlim1
Tcf21
Bnc1
Ebf1
Creb3l1
Twist1
a
Prrx1
Zfhx4
Alx3
Bcl11a
Ebf3
Alx1
Maml3
Twist2
Ankrd1
Actn2
Mlip
Mef2c
Smyd1
Fhl2
Tgfb1i1
Tbx20
Osr1
Foxf1
Tshz1
Gata5
8
6
4
2
0
Endothelium
Epicardium/ Mesothelium
Mesodermal
Myocardium
CPP
B
12.5
Ldb2
Fli1
Lmo2
Elk3
Sox17
Sox18
Erg
Klf2
Tcf15
Hdac9
Wt1
Aebp1
Sox6
Tbx18
Myrf
Npas3
Atf3
Zdbf2
Hand2
Creb3
Tcf21
Trps1
Snai2
Tbx4
Gli3
Hoxb2
Aff3
Maml3
Actn1
Pbx3
Actn2
Mlip
Ankrd1
Fhl2
Lrrfip1
Cited1
Smyd1
Ppargc1a
Mitf
Esrrg
8
6
4
2
0
Endothelium
Epicardium/ Mesothelium
Mesodermal
Myocardium
C
17.5
Ldb2
Fli1
Elk3
Sox17
Sox18
Erg
Lmo2
Mrtfb
Hdac9
Epas1
Wt1
Aebp1
Sox6
Myrf
Zbtb7c
Atf3
Zdbf2
Creb3l1
Tbx18
Tob1
Meg3
Tcf21
Basp1
Cebpb
Tbx4
Egr1
Rora
Meis1
Tshz2
Sox5
Actn2
Fhl2
Mlip
Cited1
Smyd1
Ankrd1
Ppargc1a
Tbx20
Mitf
Ss18l2
8
6
4
2
0
Endothelium
Epicardium Mesothelium
Mesodermal
Myocardium

Fig. S6. Differential effector gene expression in *Wnt2*+ CPP-derived progeny across development, related to Figure 6. (A-C) Heat maps demonstrating differential transcription factor and effector gene expression in cell compartments of heart and lung at (A) E10.5, (B) E12.5, and (C) E17.5.

Figure S7

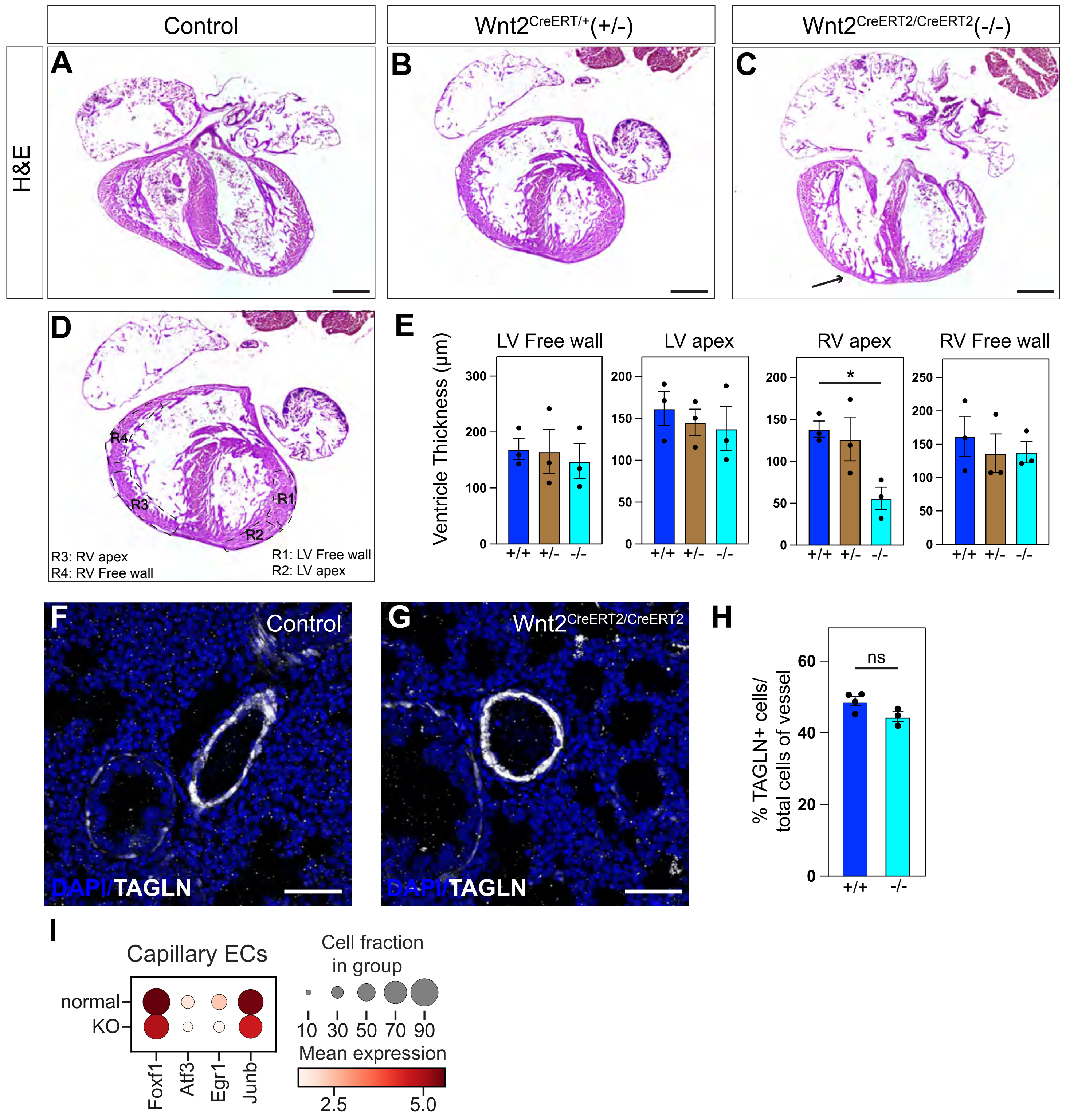

Control
Wnt2CreERT/+(+/-)
Wnt2CreERT2/CreERT2(-/-)
H&E
A
B
C
D
R1
R2
R3
R4
R3: RV apex
R4: RV Free wall
R1: LV Free wall
R2: LV apex
E
Ventricle Thickness (μm)
LV Free wall
LV apex
RV apex
RV Free wall
+/+ +/- -/-
F
Control
DAPI/TAGLN
G
Wnt2CreERT2/CreERT2
DAPI/TAGLN
H
% TAGLN+ cells/ total cells of vessel
ns
+/+ -/-
I
Capillary ECs
normal
KO
Foxf1
Atf3
Egr1
Junb
Cell fraction in group
10 30 50 70 90
Mean expression
2.5 5.0

Fig. S7. Ventricular hypoplasia in homozygous null *Wnt2* mice, related to Figure 7. (A-C) Hematoxylin and eosin (H&E) of E17.5 hearts from (A) wild type, (B), heterozygous *Wnt2*$^{CreERT2/+}$, and (C) homozygous *Wnt2*$^{CreERT2/CreERT2}$ mice. Black arrow indicates area of ventricular hypoplasia. (D) Representative H&E identifying different ventricular regions. (E) Thickness measurements of LV free wall, LV apex, RV apex and RV free wall, n=3 embryos. Data are represented as mean +/- SEM. *$p < 0.05$; Students t-test. (F-G) IHC for TAGLN in (F) control and (G) homozygous *Wnt2*$^{CreERT2/CreERT2}$ E17.5 lungs. (H) Percent of TAGLN+ cells in blood vessel, n=3. Data are represented as mean +/- SEM. p = ns; Students t-test. (I) Dot plots of representative endothelial genes downregulated in EYFP+ ECs of *Wnt2*$^{CreERT2/+}$ and *Wnt2*$^{CreERT2/CreERT2}$ E17.5 lungs. Scale bar: 500 µm for A-C, 50 µm for F-G.

Table S1. Genes up in ECs from heterozygous *Wnt2*$^{CreERT2/+}$ lungs at E17.5, related to Figure 7.

names	scores	logfoldchanges	pvals_adj
Stmn1	30.580095	4.9914274	4.5737E-201
Rbp1	19.026314	3.248673	6.99213E-77
Ier3	17.0935	2.6267662	8.42527E-62
Cldn5	15.5170145	1.1686894	7.72331E-51
Cdk14	15.162403	2.3959758	1.59282E-48
Ier2	14.979281	1.3233528	2.26368E-47
Cmtm8	13.958627	2.7691138	5.14759E-41
Junb	13.06653	1.3751447	7.99167E-36
Cebpd	12.638743	1.552816	1.87316E-33
Socs3	12.497254	2.080183	1.04645E-32
Scn7a	12.351054	1.3122398	5.42555E-32
Pltp	10.386277	1.3163996	2.42328E-22
Ankrd37	10.146418	1.7936504	2.79304E-21
Slco2a1	10.139018	2.0139892	2.89701E-21
Fos	8.891988	1.3759582	3.48371E-16
Dnah8	8.612079	1.569866	3.73694E-15
Rabgef1	8.458975	1.0875087	1.33639E-14
Irf1	8.370141	1.3864926	2.7186E-14
Cpne8	8.355151	1.199307	3.0169E-14
Pim3	8.351565	1.0883684	3.04081E-14
Egr1	8.17447	1.5581728	1.18361E-13
Slc12a2	8.169607	1.3732423	1.20861E-13
Akap12	8.100874	1.0554687	2.05258E-13
Ccdc28b	7.9385204	1.0761001	7.16583E-13
Zfp36	7.9235115	1.4324507	7.81697E-13
Ace	7.712488	1.3575906	3.99746E-12
Scn3b	7.650087	1.4873849	6.18141E-12
Adgrl3	7.194799	1.1928256	1.6084E-10
Gadd45g	7.1863317	1.2489119	1.68992E-10
Pde4b	7.184367	1.0656807	1.69323E-10
Ppp1r15a	7.0610714	1.1528313	3.90254E-10
Arap2	6.9210124	1.0868019	9.90098E-10
Pcsk5	6.735602	1.4998269	3.41872E-09
Lyve1	6.5815444	1.0071062	8.75692E-09
Aldh2	6.476633	1.2689868	1.67122E-08
Nova1	5.5730186	1.1869192	2.95676E-06
Peg3	5.414975	1.1760489	6.73044E-06
Atf3	5.3836412	1.1155089	7.84512E-06
Prx	5.2415414	1.0952884	1.58562E-05
Csrnp1	5.196533	1.232852	1.98754E-05
Aff3	5.005058	1.1066966	4.99729E-05
Acer2	4.8906684	1.0483408	8.16535E-05
Pde3a	4.54956	1.355176	0.000358029

Mfap2	4.351486	1.1236938	0.000793838
Ryr2	4.2345605	4.3011823	0.001264039
Nkd1	3.8071165	1.0062369	0.005621923
Eif2s3y	3.6306312	29.001583	0.010022814

Table S3. Primary antibody concentrations for IHC, related to STAR Methods section

Antibody	Concentration
Rabbit anti-ESRRG	1:100
Rabbit anti-SOX6	1:50
Goat anti-GFP	1:100
Chicken anti-GFP	1:100
Mouse anti-ACTA2	1:100
Goat anti-TAGLN	1:100
Rabbit anti-VWF	1:100
Goat anti-EMCN	1:200
Rat anti-EMCN	1:100
Mouse anti-ERG	1:100
Rabbit anti-WT1	1:50
Mouse anti-TNNT2	1:200
Rat anti-PECAM1	1:200
Goat anti-PDGFRB	1:200
Rabbit anti-PDGFRA	1:200
